# Pangenomic analysis identifies structural variation associated with heat tolerance in pearl millet

**DOI:** 10.1038/s41588-023-01302-4

**Published:** 2023-03-02

**Authors:** Haidong Yan, Min Sun, Zhongren Zhang, Yarong Jin, Ailing Zhang, Chuang Lin, Bingchao Wu, Min He, Bin Xu, Jing Wang, Peng Qin, John Pablo Mendieta, Gang Nie, Jianping Wang, Chris S. Jones, Guangyan Feng, Rakesh K. Srivastava, Xinquan Zhang, Aureliano Bombarely, Dan Luo, Long Jin, Yuanying Peng, Xiaoshan Wang, Yang Ji, Shilin Tian, Linkai Huang

**Affiliations:** 1 College of Grassland Science and Technology, Sichuan Agricultural University, Chengdu, China; 2 School of Plant and Environmental Sciences, Virginia Tech, Blacksburg, VA USA; 3 Department of Genetics, University of Georgia, Athens, GA USA; 4 Novogene Bioinformatics Institute, Beijing, China; 5 State Key Laboratory of Crop Gene Exploration and Utilization in Southwest China, Sichuan Agricultural University, Chengdu, China; 6 College of Grassland Science, Nanjing Agricultural University, Nanjing, China; 7 Key Laboratory of Bio-Source and Environmental Conservation, School of Life Science, Sichuan University, Chengdu, China; 8 Rice Research Institute, Sichuan Agricultural University, Chengdu, China; 9 Agronomy Department, University of Florida, Gainesville, FL USA; 10 Feed and Forage Development, International Livestock Research Institute, Nairobi, Kenya; 11 International Crops Research Institute for the Semi-Arid Tropics, Hyderabad, India; 12 Instituto de Biologia Molecular y Celular de Plantas, UPV-CSIC, Valencia, Spain; 13 College of Animal Science and Technology, Sichuan Agricultural University, Chengdu, China; 14 Triticeae Research Institute, Sichuan Agricultural University, Chengdu, China; 15 Sichuan Animal Science Academy, Chengdu, China; 16 Department of Ecology, Hubei Key Laboratory of Cell Homeostasis, College of Life Sciences, Wuhan University, Wuhan, China

**Keywords:** Genomics, Plant genetics

## Abstract

Pearl millet is an important cereal crop worldwide and shows superior heat tolerance. Here, we developed a graph-based pan-genome by assembling ten chromosomal genomes with one existing assembly adapted to different climates worldwide and captured 424,085 genomic structural variations (SVs). Comparative genomics and transcriptomics analyses revealed the expansion of the RWP-RK transcription factor family and the involvement of endoplasmic reticulum (ER)-related genes in heat tolerance. The overexpression of one *RWP-RK* gene led to enhanced plant heat tolerance and transactivated ER-related genes quickly, supporting the important roles of RWP-RK transcription factors and ER system in heat tolerance. Furthermore, we found that some SVs affected the gene expression associated with heat tolerance and SVs surrounding ER-related genes shaped adaptation to heat tolerance during domestication in the population. Our study provides a comprehensive genomic resource revealing insights into heat tolerance and laying a foundation for generating more robust crops under the changing climate.

## Abstract (web summary)

A graph-based pan-genome constructed using de novo genome assemblies of ten pearl millet accessions adapted to different climates worldwide identifies structural variations and their contribution to heat tolerance in pearl millet.

## Main

Global warming has severely affected crop productivity, which seriously threatens world food security^1^. The change in temperature from the historical average in 1900 is expected to exceed 2 °C by the end of the twenty-first century^2^. With every 1 °C increase in the global average temperature, wheat (*Triticum aestivum*) production is estimated to decrease by 6%, rice (*Oryza sativa*) production is estimated to decrease by 3.2% and corn (*Zea mays*) production is estimated to decrease by 7.4%^3^. Therefore, an understanding of heat tolerance in plants is urgently required to develop crops that can withstand rising global temperatures and could thus be used to maximize agricultural production to help satisfy the food demands of an increasing population.

Pearl millet (*Pennisetum glaucum* (L.) R. Br., syn. *Cenchrus americanus* (L.) Morrone) (2*n* = 2*x* = 14) is a C_4_ cereal crop that is important in safeguarding the security of food and forage in the arid and semiarid tropics due to its superior tolerance to high temperatures^4–8^. It is also a staple food of more than 90 million farmers living in poverty and is grown on more than 31.2 million hectares^9^. Pearl millet is an ideal model for understanding how plants use heat-related genes and mechanisms to thrive at warmer temperatures. However, few studies have investigated the molecular mechanisms underlying the regulation of heat stress responses (HSRs) in pearl millet relative to other major crops^10,11^ and the underlying mechanisms are not well understood.

Recent studies revealed that many genes involved in environmental stress responses are strongly affected by structural variations (SVs)^12–14^; however, the causal relationship of SVs with HSRs is poorly understood. SVs have roles in gene expression alterations linked to important plant phenotypes^15^. However, the detection of SVs is challenging when relying on short-read sequencing data^16,17^. This challenge has promoted the development of new approaches for SV detection using graph-based pan-genomes that are based on multiple high-quality assemblies^17–19^. Therefore, building graph-based pan-genomic resources has the potential to advance the characterization and understanding of the biological impact of SVs on phenotypic variations and accelerate the breeding of pearl millet.

In this study, we generated de novo genome assemblies of ten pearl millet accessions and constructed a graph-based pan-genome assembly to identify genomic SVs. We leveraged SVs, transcriptomics and in vivo validation to reveal the relationship between SVs and gene expression under heat stress conditions. With this approach, we identified SVs that contributed to heat adaptation during crop domestication. By integrating multi-omics analyses, we suggested a possible mechanism in which the resistance of pearl millet to heat stress depends mainly on the endoplasmic reticulum (ER) and validated an RWP-RK (https://www.ebi.ac.uk/interpro/entry/pfam/PF02042/) transcription factor as a positive coregulator of heat tolerance along with the ER pathway. Our findings advance the conceptual understanding of heat tolerance in pearl millet, promise to expedite genomics-assisted breeding for heat tolerance in this important crop and will benefit comparative and functional genomics studies of other crops.

## Results

### Genome assembly and pan-genomic analysis of representative pearl millet accessions

**Fig. 1:**
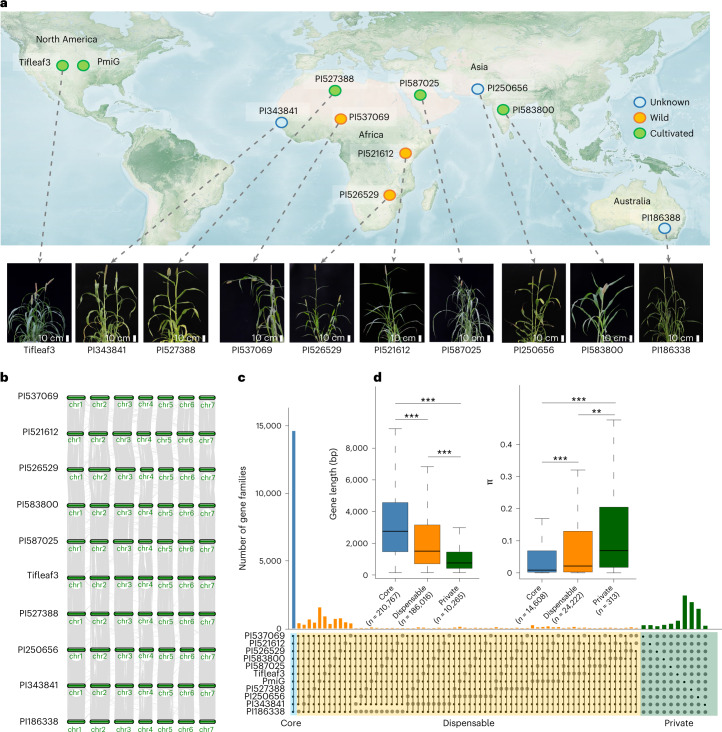
Ten high-quality assembled genomes and pan-genome construction in pearl millet. **a**, The pearl millet accessions are derived from geographically representative regions (PmiG: Tift 23D2B1-P1-P5). The geographical map was adapted from the one provided by the NASA Earth Observatory (https://visibleearth.nasa.gov/images/147190/explorer-base-map/147191w/). **b**, Synteny plot across the ten genomes. **c**, Core gene clusters and pan-genome of pearl millet. The histogram illustrates the core gene clusters (present in all genomes), dispensable gene clusters (present in 2–10 genomes) and private gene clusters (present in one genome). **d**, Composition of gene and nucleotide diversity (*pi*) in core, dispensable and private genes. The center line represents the median; the box limits represent the upper and lower quartiles; the whiskers represent 1.5 times the interquartile range (IQR). Significant differences were tested by two-tailed *t*-test (**P < 0.01, ***P < 0.0005).

**Extended Data Fig. 1:**
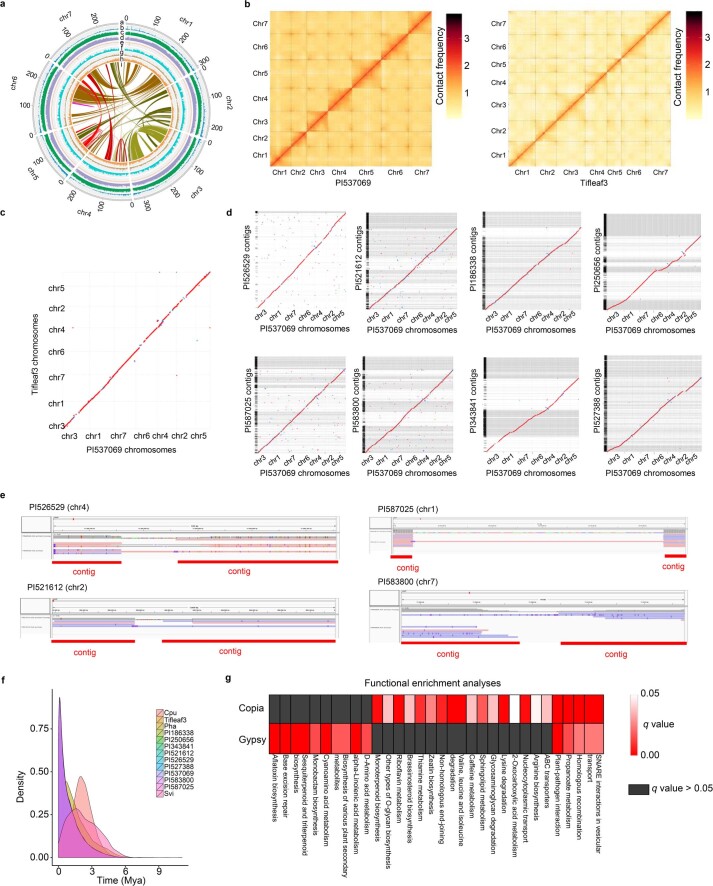
High quality assembled genomes in pearl millet. **a**, Genome landscape of pearl millet (PI537069). Track ‘a’: the seven chromosomes at the Mb scale; track ‘b’: chromosomal distribution of gene models in which the gene density ranged from 201 to 503,365 bp/Mb; track ‘c’: chromosomal distribution of TEs for which the density was 58,334 to 945,223 bp/Mb; track ‘d’: TE/LTR distribution, ranging from 122,500 to 920,183 bp/Mb; track ‘e’: GC content along the assembled genome, which ranged between 20% and 60%/Mb; track ‘f’ and ‘g’: numbers of SNPs (5 to 27,243/Mb) and indels (1 to 5,952/Mb); track ‘h’: number of SVs (6 to 422/Mb); track ‘i’: collinear blocks of the pearl millet (PI537069) genome. **b**, Hi-C contact matrices of PI537069 and Tifleaf3 assemblies. **c**, Collinearity between the PI537069 and Tifleaf3 genomes. The contigs of Tifleaf3 were connected with Hi-C data only. This plot showed good synteny of the pseudochromosomes of Tifleaf3 and another assembly based on Hi-C data from PI537069. **d**, Synteny of eight contig-level assemblies with the PI537069 chromosome-level assembly. **e**, Assessment of the contig connections based on HiFi reads. Here the four examples show a long HiFi read across two contigs. **f**, Insertion times of LTRs in pearl millet and three related species, *Cenchrus purpureus* (Cpu), *Panicum hallii* (Pha) and *Setaria viridis* (Svi). **g**, Functional enrichment analyses of genes encompassed by intact Copia and Gypsy TEs.

**Extended Data Fig. 2:**
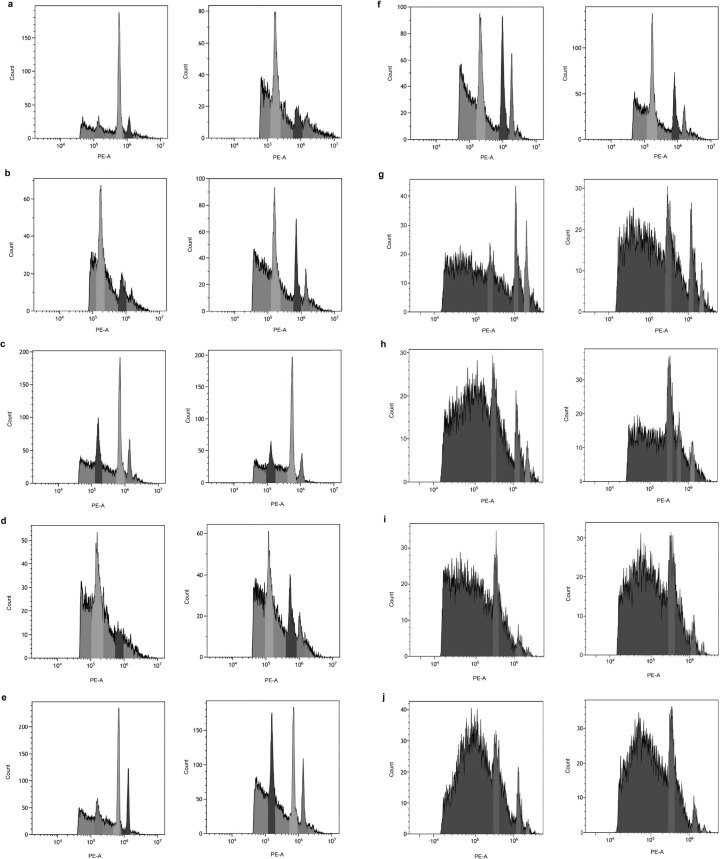
Estimation of genome size using flow cytometry. Estimation of genome size using flow cytometry. **a-j**, Histogram of relative fluorescence intensities from flow cytometric analysis of Propidium iodide (PI)-stained nuclei of Tifleaf-3, PI521612, PI526529, PI537069, PI583800, PI587025, PI186338, PI250656, PI343841, PI527388, which were isolated, stained and analyzed simultaneously (two biological repeats). The *Oryza sativa* cv. Nipponbare genome (2n = 2x = ~ 420 Mb) served as an internal reference standard. The average ratio of peak value between pearl millet to *O. sativa* was equal 4.5, hence the estimated average genome size of pearl millet was ~ 1890 Mb. Source data are provided as a Source Data file.

We selected ten representative accessions from eight major geographical regions based on the phylogenetic relationships of a 394-line core collection of pearl millet^7,20^ (Fig. 1a,b, Supplementary Figs. 1 and 2 and Supplementary Table 1). We assembled their chromosome-level genomes by integrating PacBio high-fidelity (HiFi) long-read sequences, Bionano optical mapping data, high-throughput chromosome conformation capture (Hi-C) data and Illumina paired-end sequences (Fig. 1a,b, Extended Data Fig. 1, Supplementary Table 2 and Supplementary Note 1). These genomes ranged in size from 1.89 Gb to 2.00 Gb, with scaffold N50 values ranging from 193.80 Mb to 286.98 Mb, corresponding to 95.85–99.47% of the genome sizes estimated by *k*-mer analysis (1.97–2.01 Gb), which is consistent with the genome sizes predicted by flow cytometry (Extended Data Fig. 2). The contig N50 values were substantially increased from 155 to 3,959-fold over those of the previously published pearl millet reference genome^7^ (Table 1 and Supplementary Table 2).

**Table 1:** Summary of genome assembly and annotation

Accession no.	Contig N50 (Mb)	Scaffold N50 (Mb)	Contig length (Mb)	Scaffold length (Mb)	Chromosome anchoring rate (%)	Repeat ratio (%)	Gene no.	LAI
PI537069	61.62	266.84	1,908.34	1,913.80	96.68	71.58	35,486	27.90
PI521612	5.40	278.46	1,891.01	1,891.08	95.98	70.44	37,906	26.15
PI587025	5.15	257.50	1,911.08	1,911.21	94.39	71.58	38,076	27.38
PI583800	3.10	261.45	1,937.87	1,937.98	97.52	72.21	35,826	27.53
Tifleaf3	25.57	279.17	1,950.21	1,950.23	95.00	71.30	37,280	26.22
PI526529	79.18	286.98	1,974.39	1,974.39	98.48	71.88	36,451	26.53
PI186338	3.80	284.64	1,999.44	1,999.53	95.30	72.63	36,343	26.47
PI343841	5.10	263.66	1,962.06	1,962.24	94.23	72.17	36,312	26.76
PI527388	3.10	193.80	1,937.79	1,938.01	94.51	71.02	37,866	27.79
PI250656	4.20	276.63	1,895.51	1,895.77	95.11	70.72	36,923	24.74
Tift 23D2B1-P1-P5/PmiG^7^	0.02	0.88	1,556.18	1,793.24	NA	77.20	38,579	2.09

NA, not applicable.

To measure the quality of these ten newly assembled genomes, we realigned high-quality paired-end reads against the assemblies and observed alignment rates ranging from 95.62% to 99.57%, covering 94.92–99.90% of the genomes (Supplementary Table 2). Additionally, more than 91.60% of the embryophyte Benchmarking Universal Single-Copy Orthologs (BUSCOs) were present in each genome (Supplementary Table 2). The long terminal repeat (LTR) assembly index (LAI) scores all exceeded 24 and thus met the criterion standard^21^ (Table 1). Further evaluation using Merqury showed a quality value (QV) over 40 for our ten assemblies, which exceeded the Vertebrate Genomes Project standard of QV40^22^ (Supplementary Table 2). These results demonstrate the accuracy, completeness and contiguity of the ten pearl millet genome assemblies. In addition, we predicted an average of 36,847 gene models for each assembly, among which more than 99.30% showed matches with the known functional database (Supplementary Table 2). Transposable elements (TEs) constituted 71.58% of each genome, ranging from 70.44% to 72.62% (Supplementary Tables 2–4 and Supplementary Note 1).

We constructed a pan-genome using 11 pearl millet assemblies, including the previously released genome^7^. Among the total gene family sets, 14,608 core gene families were obtained across all accessions, accounting for more than half (46.60–52.08%) of the total sets; dispensable families (39.75–49.94%), in which genes were present in 2–10 accessions, constituted the second-largest proportion. The smallest proportion consisted of private gene sets, which were only detected in one genome and accounted for 0.73–8.73% of the total sets (Fig. 1c).

**Extended Data Fig. 3:**
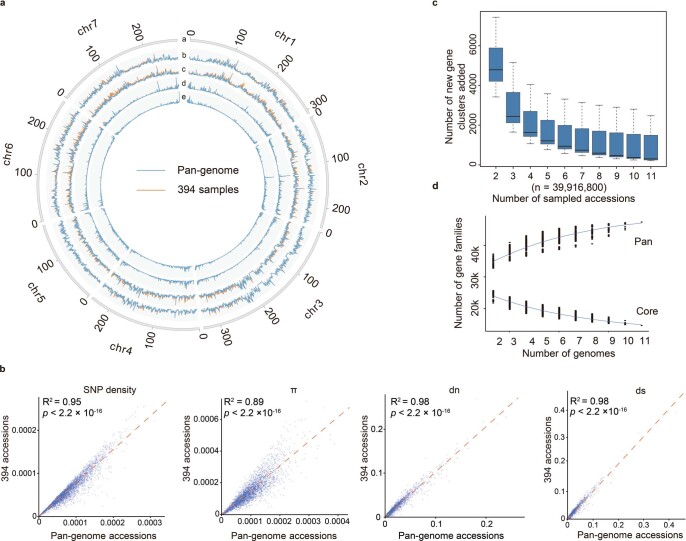
Evaluation of the representativeness of the pearl millet pan-genome. **a**, Distribution of genetic variations from 11 genomes and 394 re-sequenced pearl millet accessions. Track ‘a’: the seven chromosomes at the Mb scale; track ‘b’: SNP density; track ‘c’, ‘d’, and ‘e’ represent nucleotide diversity (π), nonsynonymous (d_N_), and synonymous (d_S_) substitution rates, respectively. **b**, Correlation of SNP density, π, d_N_, and ds from the 11 de novo assembled genomes and the 394 re-sequenced accessions. Significant differences were tested by two-tailed Pearson correlation test and shown by *p* value. **c**, Number of newly added gene families when adding more accessions. The median number of new gene families is 301 when the last accession comes in. Center line, median; box limits, upper and lower quartiles; whiskers, 1.5x interquartile range. **d**, The variation in gene families in the core and pan-genomes along with additional pearl millet genomes. Source data

**Extended Data Fig. 4:**
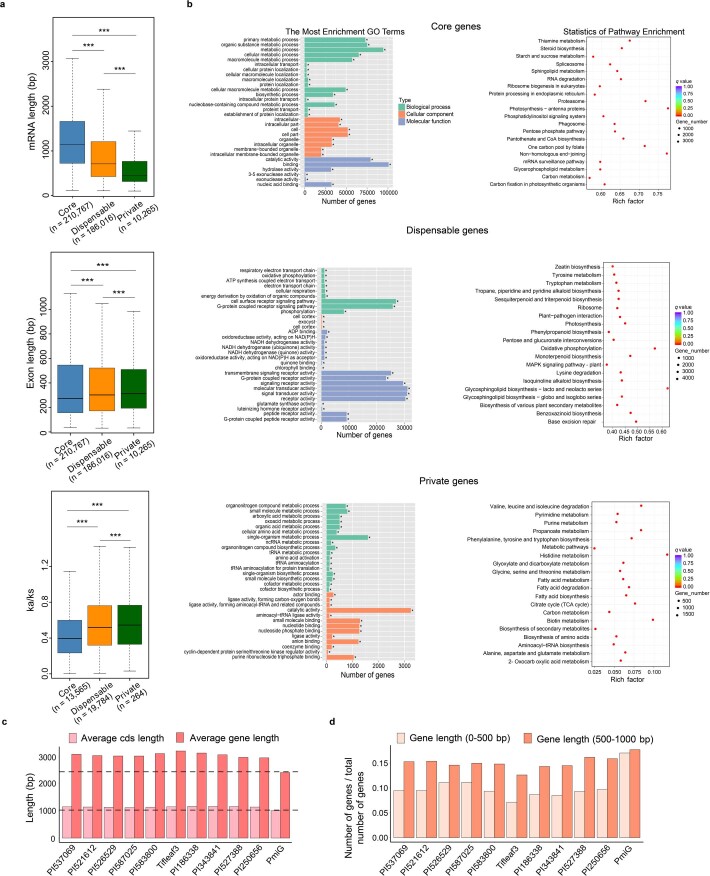
Characterization of core, dispensable, and unique gene sets. **a**, mRNA length, exon length, and ka/ks values of the three gene categories. Center line, median; box limits, upper and lower quartiles; whiskers, 1.5x interquartile range; and dots represent outliers. Significant differences were tested by two-tailed *t*-test (***P < 0.0005). **b**, Gene Ontology (GO) (left) and Kyoto Encyclopedia of Genes and Genomes (KEGG) (right) enrichment results. Significant differences were tested by hypergeometric test (*FDR-adjusted *P* < 0.05). **c**, Comparison of average CDS and gene length across all accessions. The dotted lines indicate average CDS and gene lengths in the PmiG genome. **d**, Comparison of proportion of genes in two length ranges across all accessions.

To further evaluate the representativeness of the pan-genome, we compared the distribution of SNPs between the 11 accessions and the aforementioned 394 core lines. They displayed a similar pattern across the genome and showed strong significant correlations in SNP density, nucleotide diversity (*π*) and synonymous (*d*_S_) and nonsynonymous (*d*_N_) substitution rates (SNP density, *rho* = 0.95; *π*, *rho* = 0.89; *d*_S_, *rho* = 0.98; *d*_N_, *rho* = 0.98) (Extended Data Fig. 3a,b). The number of added gene families declined quickly, with only 301 (0.64% of all gene families; 301 out of 47,344) additional gene families being identified when the eleventh accession was included (Extended Data Fig. 3c,d). Moreover, the accessions used to generate the pan-genome showed a similar Shannon’s diversity index (*H*) and *π* to the 394 accessions (*H*: 8.07782 versus 8.03436; *π*: 0.0001327 versus 0.0001209). In general, these results suggest that the pan-genome accessions are genetically diverse and representative of the diversities of the pearl millet population. We further observed that core genes were more functionally conserved and enriched in general biological processes than the dispensable and private genes, as with previous findings in other plants^17,23,24^ (Fig. 1d, Extended Data Fig. 4 and Supplementary Note 2). In total, we built a high-quality pan-genome resource that will contribute to pearl millet improvement.

### Graph-based genome and SV identification

**Fig. 2:**
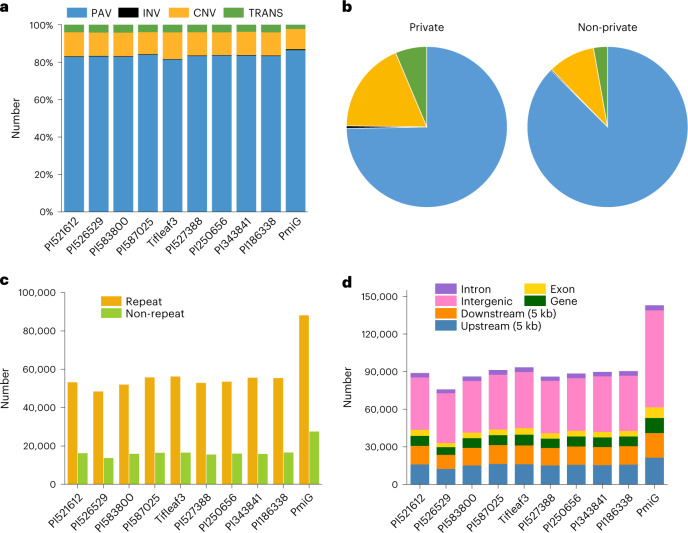
Identification of SVs. **a**, Composition of SVs in each genome. **b**, Comparisons of the proportions of private (present in only one accession) and non-private SVs (present in at least two accessions). **c**, Numbers of SVs in the repeat and non-repeat regions of each genome. **d**, Numbers of SVs overlapping with different genomic features in each genome.

**Extended Data Fig. 5:**
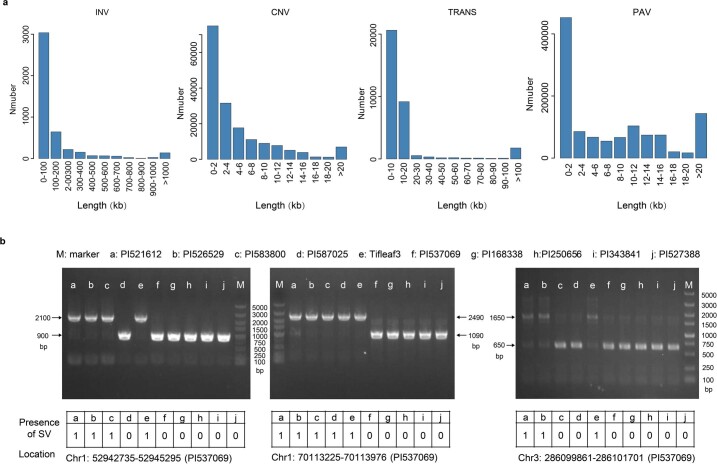
Characterization of structural variations (SVs). **a**, Length distributions of four major SV types. PAV, TRANS, CNV, INV: presence and absence variation, translocation, copy number variation, and inversion, respectively. **b**, PCR validation of three SVs in 10 pearl millet accessions. Each experiment is performed once. The images were cropped from the images in the Source data file. Source data are provided as a Source Data file. Source data

A total of 744,364 SVs were identified by realigning the assemblies against the PI537069 reference genome as this accession comes from the geographical origin (Northwest Africa) of pearl millet^25^ and has a relatively high assembly quality (Table 1 and Supplementary Table 2). These SVs included 622,584 presence and absence variations (PAVs) consisting of 306,679 presence and 315,905 absence cases, 2,177 inversions (INVs), 91,852 copy number variations (CNVs) and 27,751 translocations (TRANS) (Fig. 2a and Supplementary Table 5). Approximately 37.94% of PAVs were less than 2 kb in length, INVs (68.11%) were concentrated within 100 kb, CNVs (62.53%) were enriched in the size range of less than 4 kb and most TRANS (91.10%) were less than 20 kb in length (Extended Data Fig. 5a).

To build the graph-based genome, the SVs from all the pearl millet accessions were merged to yield 424,085 non-redundant SVs. PAVs accounted for 74.70% of private SVs present in only one accession but constituted a relatively high proportion (87.51%) of the non-private SVs. Similar trends were observed for CNVs and TRANS (Fig. 2b). We observed that the SVs were enriched in repeat regions (Fig. 2c). Across these genomes, 37–44% of SVs overlapped with genic and flanking regions (5 kb) (Fig. 2d), suggesting potential roles of SVs in gene regulation. In addition, the SVs and graph-based genome were validated by evaluating the performances of different SV calling tools, by conducting PCR and checking read coverage over the possible variant paths (Extended Data Fig. 5b, Supplementary Tables 6–8 and Supplementary Note 3). Overall, this graph-based pan-genome is an essential genomic resource supporting the study of SVs and will provide a prominent reference for the discovery of SVs in pearl millet populations.

### Expansion of the RWP-RK transcription factor family contributes to heat tolerance

**Fig. 3:**
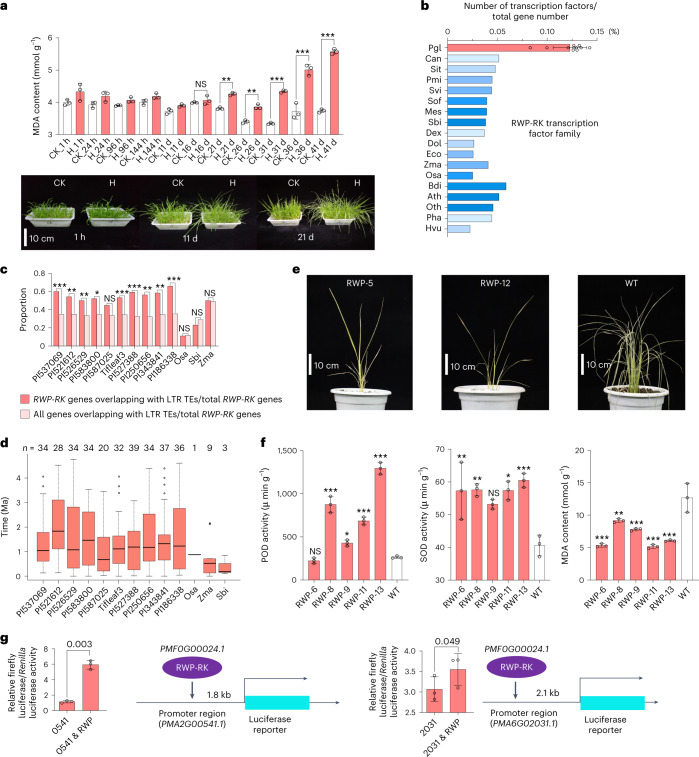
Expansion of the RWP-RK transcription factor family contributes to heat tolerance. **a**, Comparison of MDA levels between the control (CK) and heat treatment (H) groups of pearl millet (Tifleaf3). The error bars indicate the mean ± s.d.; *n* = 3 biological replicates. Significant differences were tested by two-tailed *t*-test (**P < 0.01, ***P < 0.0005; NS, not significant). **b**, Proportion of RWP-RK transcription factor family members among all the genes in the 11 pearl millet genomes and ten other genomes. Ath, *Arabidopsis thaliana*; Bdi, *Brachypodium distachyon*; Can, *Capsicum annuum*; Dex, *Digitaria exilis*; Dol, *Dichanthelium oligosanthes*; Eco, *Eleusine coracana*; Hvu, *Hordeum vulgare*; Mes, *Manihot esculenta*; Osa, *O. sativa*; Oth, *Oropetium thomaeum*; Pgl, *P. glaucum* (pearl millet); Pha, *Panicum hallii*; Pmi, *Panicum miliaceum*; Sbi, *Sorghum bicolor*; Sit, *Setaria italica*; Sof, *Saccharum officinarum*; Svi, *Setaria viridis*; Zma, *Z. mays*. **c**, Comparisons of *RWP-RK* gene numbers overlapping with intact LTR TEs among pearl millet, rice, sorghum and maize. Significant differences were tested by one-tailed binomial test (*P < 0.05, **P < 0.01, ***P < 0.0005). **d**, Estimated insertion times of LTR TEs encompassing the *RWP-RK* genes shown in **c**. The center line represents the median; the box limits represent the upper and lower quartiles; the whiskers represent 1.5 times the IQR; the dots represent the outliers. **e**, Phenotypes of two transgenic lines and a control (WT) after 72 h of heat treatment. **f**, POD and SOD activity after 12 h of heat treatment and MDA content after 72 h of heat treatment in transgenic lines and WT plants. The error bars represent the mean ± s.d.; *n* = 3 biological replicates. Significant differences were tested using a two-tailed *t*-test (*P < 0.05, **P < 0.01, ***P < 0.0005). **g**, Dual luciferase assays (firefly luciferase to *Renilla* luciferase ratio) were applied to verify that *PMF0G00024.1* (RWP) could transactivate *PMA2G00541.1* (541) and *PMA6G02031.1* (2031). The error bars represent the mean ± s.d.; *n* = 3 biological replicates. Significant differences were tested using a two-tailed *t*-test and are shown as *P* values.

**Extended Data Fig. 6:**
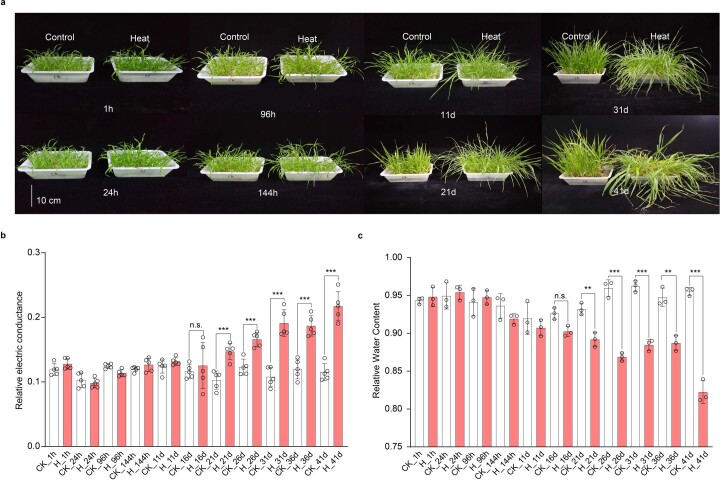
Phenotypes and physiological indicators under heat stress conditions. **a**, Phenotypic changes in pearl millet (Tifleaf3) under heat stress (40°C/35°C) at eight time points. Scale bar indicates 10 cm. **b-c**, Relative conductance (b) and relative water content (c) of the Tifleaf3 accession at 11 time points under control and heat treatments. Error bars, mean ± s.d.; n = 5 biological replicates for b, and n = 3 biological replicates for c. Significant differences were tested by two-tailed *t*-test (*P < 0.05, **P < 0.01, ***P < 0.0005; n.s., not significant).

Pearl millet was shown to be very tolerant to high-temperature conditions based on our phenotypic and physiological data (Fig. 3a). In particular, the leaves of pearl millet seedlings only showed wilting after 21 d of heat treatment (40 °C in light, 35 °C in darkness) (Extended Data Fig. 6a). The relative water content, relative electrical conductivity (REC) and malondialdehyde (MDA) content did not change significantly (*P* > 0.05) until 21 d of heat treatment (Extended Data Fig. 6b,c), while in maize leaves, the relative water content decreased and the MDA content increased significantly under 4 h of heat stress (40 °C)^26^. The slower responses might indicate better heat tolerance in pearl millet than in maize.

**Extended Data Fig. 7:**
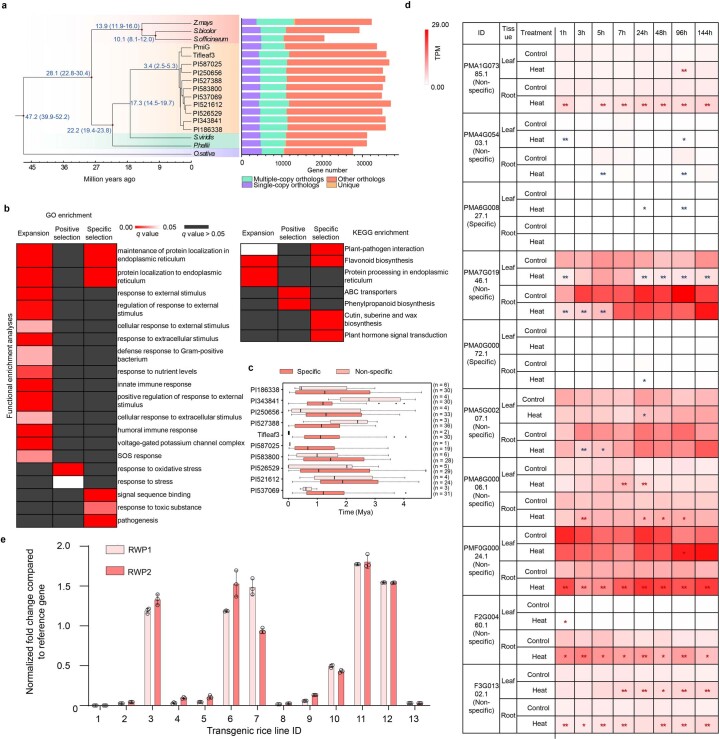
Expansion and expression of RWP-RK transcription factor (TF) family members. **a**, Estimation of the divergence times of seven pearl millet accessions and six closely related species. PmiG represents Tift 23D2B1-P1-P5. The right panel displays the distribution of single-copy, multiple-copy, unique and other gene orthologs. **b**, Functional enrichment analyses showed the enrichment of some genes in stress-related processes or pathways among expanded family, specific, and positively selected gene sets. **c**, Estimated insertion times of LTR TEs encompassing the specific RWP-RKs of pearl millet belonging to clade A and the nonspecific RWP-RKs belonging to clade B. Center line, median; box limits, upper and lower quartiles; whiskers, 1.5x interquartile range; and dots represent outliers. **d**, Expression of seven RWP-RK genes under control (CK) and heat treatments (H). Significant differences were performed by DESeq2 to determine fold change (FC) of gene expressions between the CK and H groups (two-tailed Wald test). The FC was determined by * (0 < |log2FC|< 1, FDR-adjusted *P* < 0.05) or ** (|log2FC|>= 1, FDR-adjusted *P* < 0.05). The blue and red */** represent gene down- and upregulation, respectively. **e**, Expression of two RWP fragments (RWP1 and RWP2) in 13 transgenic rice lines. A value above 0 indicates the upregulation of this gene. Error bars, mean ± s.d.; n = 3 biological replicates.

To dissect the molecular mechanism underlying heat tolerance in pearl millet, we first conducted comparative genomic analyses, which revealed that expanded, positively selected and species-specific gene families, as well as genes located near recently expanded LTR TEs (LTRs) were enriched in stress-related pathways in pearl millet (Extended Data Figs. 2g and 7a,b and Supplementary Note 4). Notably, one transcription factor family (RWP-RK) was identified as expanding in the genomes of the 11 pearl millet accessions (Fig. 3b, Supplementary Fig. 3 and Supplementary Table 9). This family responded to biotic or abiotic stresses^27–30^, supporting the potential roles of its members in heat tolerance. We investigated LTRs located near the *RWP-RK* genes and found that early LTR expansion might be associated with RWP-RK transcription factor family expansion and probably caused increases in specific *RWP-RK* genes in pearl millet (Fig. 3c,d, Extended Data Fig. 7c, Supplementary Fig. 3 and Supplementary Note 5).

To further characterize the roles of *RWP-RK* genes in response to heat stress, we sequenced leaf and root transcriptomes after high-temperature treatment (Supplementary Table 3). A total of ten differentially expressed *RWP-RK* genes were predicted, including two specific and eight nonspecific transcription factors (Extended Data Fig. 7d, Supplementary Table 10 and Supplementary Note 5). When overexpressing an *RWP-RK* (*PMF0G00024.1*) in rice, we found that the leaves of the transgenic lines (*RWP-RKox*) were less withered than the leaves of wild-type (WT) plants under high temperature (Fig. 3e and Extended Data Fig. 7e). The *RWP-RKox* plants showed significantly higher peroxidase (POD) and superoxide dismutase (SOD) activities and lower MDA contents after exposure to heat stress conditions than the WT plants (Fig. 3f), which provides a potential avenue for the future molecular breeding of heat-tolerant crops. We also characterized this RWP-RK transcription factor in a coregulated network and used a dual luciferase assays to verify that this transcription factor could transactivate two stress-related genes, *PMA2G00541.1* and *PMA6G02031.1* (Fig. 3g, Supplementary Table 11 and Supplementary Note 5). Taken together, these results indicate that the expansion of the RWP-RK transcription factor family has potentially contributed to heat tolerance in pearl millet.

### *RWP-RK* coregulates a fast heat response with ER-related genes

**Fig. 4:**
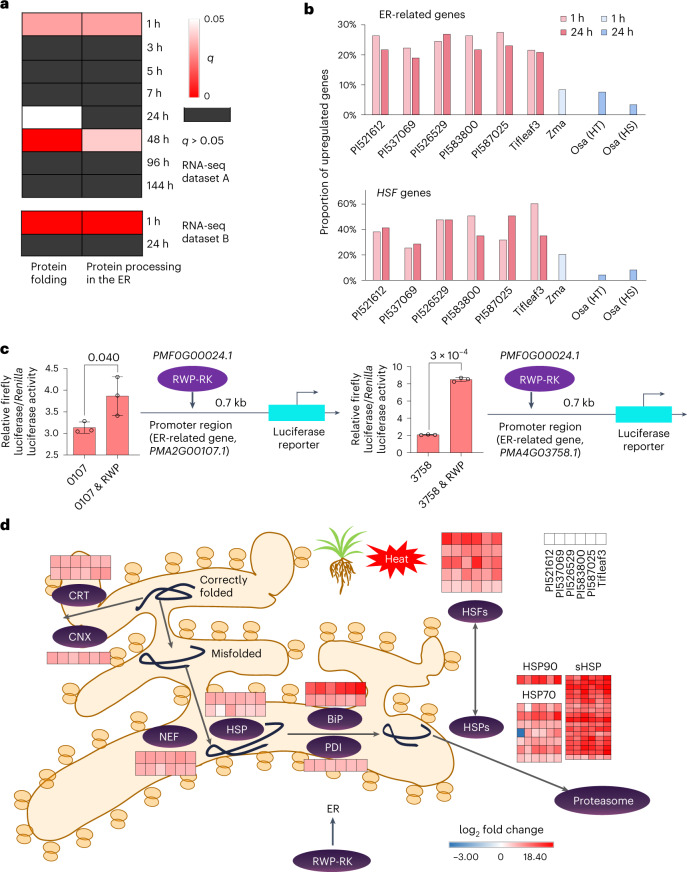
Transcriptome analyses reveal that pearl millet responds to heat stress via ER-related pathways. **a**, Functional enrichment of DEGs coexisting in Tifleaf3 under high temperature stress (40 °C and 35 °C) at eight time points (1–144 h; dataset A in Supplementary Table 3) and in six accessions (PI521612, PI537069, PI526529, PI583800, PI587025 and Tifleaf3) under high temperature stress (45 °C under light and 40 °C in darkness) for 1 h and 24 h (dataset B in Supplementary Table 3). **b**, Comparison of the proportions of upregulated ER-related and *HSF* genes after 1 h and 24 h of heat treatment in pearl millet, maize and rice. Heat-resistant (HR) and heat-susceptible (HS) rice samples, respectively. **c**, Dual luciferase assays were applied to verify that *PMF0G00024.1* (RWP) could transactivate the *PMA2G00107.1* (107) and *PMA4G03758.1* (3758) genes. The error bars represent the mean ± s.d.; *n* = 3 biological replicates. Significant differences were tested using a two-tailed *t*-test and are shown as *P* values. **d**, Proposed activation network of pearl millet in response to growth under heat stress. After 1 h of high-temperature stress in six pearl millet accessions, many misfolded proteins activated the expression of degradation-related genes in the ER, such as genes encoding recognition proteins, including calnexin (CNX) and calreticulin (CRT), and degradation-related proteins, including heat shock proteins (HSPs), thereby correcting or degrading misfolded proteins to maintain protein homeostasis in cells. In addition, the *HSF* and *RWP-RK* genes potentially participate in this process to coregulate *HSP* genes.

**Extended Data Fig. 8:**
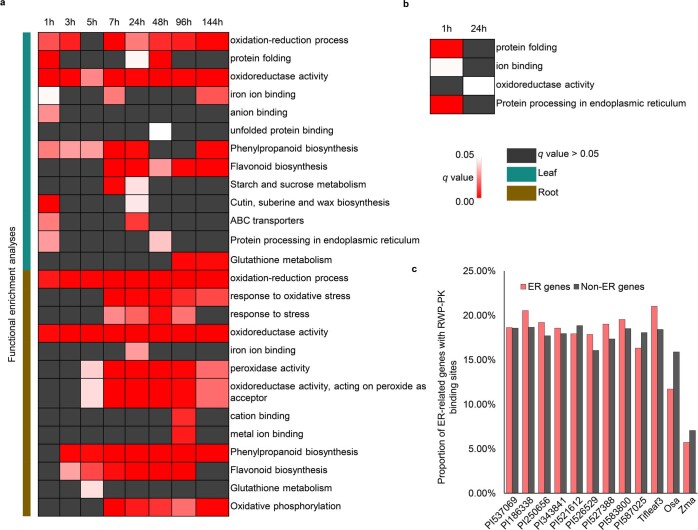
Gene functional enrichment analyses in transcriptome analyses. **a**, Functional enrichment analyses of differentially expressed genes (DEGs) identified in all six pearl millet materials after heat stress. **b**, Functional enrichment analyses of DEGs under continuous heat treatment at eight time points. **c**, Proportions of ER-related and non-ER-related genes in which RWP-RK binding sites are found in the promoter region (3 kb).

To further dissect the molecular mechanism underlying heat tolerance in pearl millet, we sequenced the leaf and root transcriptomes of Tifleaf3 under high-temperature treatments at eight time points (dataset A) and selected six accessions to perform leaf transcriptome sequencing under stress for 1 and 24 h (dataset B; Supplementary Table 3). Based on gene functional enrichment analyses, the two transcriptome datasets revealed differentially expressed genes (DEGs) that were enriched mainly in ER-related pathways involved in the repair and elimination of misfolded proteins (Fig. 4a, Extended Data Fig. 8a,b, Supplementary Table 12 and Supplementary Note 6.1). We analyzed the RNA sequencing (RNA-seq) data from maize^31^ and rice^32^ and identified greater proportions of upregulated ER-related and heat shock factor (HSF) (https://www.ebi.ac.uk/interpro/entry/pfam/PF00447/) genes in pearl millet than in these two crops under heat treatment (1 h and 24 h; Fig. 4b).

In addition, the aforementioned ten *RWP-RK* genes exhibited significant correlations (Pearson’s *rho* ≥ 0.6, *P* < 0.05) with most ER-related genes (60.2%; 325 out of 540) and *HSF* genes (50%; 16 out of 32) in response to heat stress (Supplementary Table 12), suggesting that *RWP-RK* genes might coregulate the heat tolerance of pearl millet with some ER-related genes and *HSF* genes. We further predicted potential RWP-RK binding sites upstream of these genes and found that higher proportions of ER-related genes had binding sites in pearl millet than in maize and rice (Extended Data Fig. 8c). The transient coexpression of the aforementioned *RWP-RK* (*PMF0G00024.1*) and two ER-related genes, encoding an immunoglobulin protein (BiP) (https://www.kegg.jp/entry/K09490; *PMA2G00107.1*) and the oligosaccharyltransferase complex (OST) (https://www.kegg.jp/entry/K12669; *PMA4G03758.1*), further confirming that *RWP-RK* functions at least partially by transactivating ER-related genes (Fig. 4c). Collectively, these results indicate that pearl millet may quickly respond to heat stress at the gene transcription level via the coregulation of *RWP-RK* genes with *HSF* genes and ER-related genes to eliminate proteins with temperature-induced misfolding (Fig. 4d).

### Several focal SVs are associated with heat-related gene expression

**Fig. 5:**
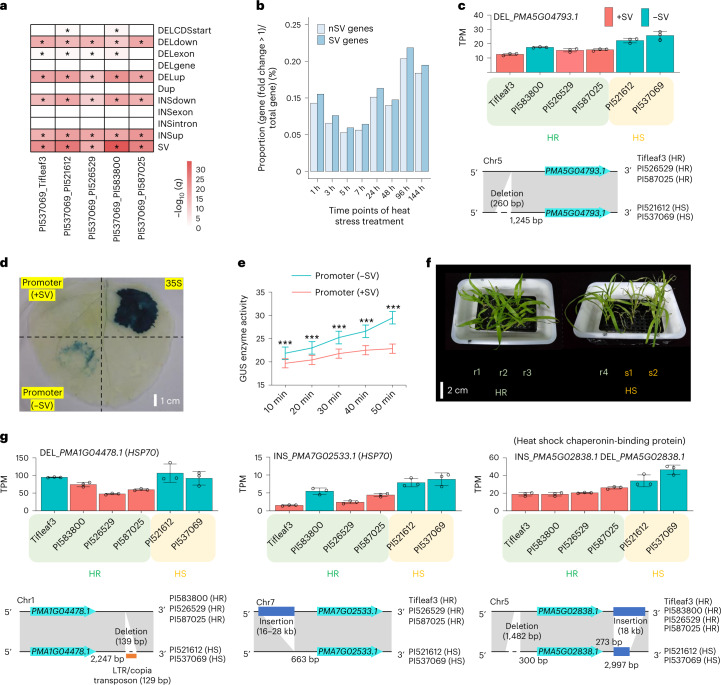
Impact of SVs on genes and their contributions to heat tolerance in pearl millet. **a**, Enrichment of SVs near genes with altered expression in each accession relative to PI537069. The asterisk indicates significance (*q* < 0.05). DEL, deletion; Dup, duplication; INS, insertion. **b**, Proportions of DEGs among total genes overlapping with SVs (SV genes) and those not overlapping with SVs (nSV genes) in leaf tissue under heat treatment. **c**, Deletion near *PMA5G04793.1* with expression changes in different accessions. TPM, transcripts per million. +SV and −SV: accessions with and without SVs, respectively. The error bars indicate the mean ± s.d.; *n* = 3 biological replicates. **d**,**e**, Transformation of the *PMA5G04793.1* promoter in tobacco leaves. **d**, Glucuronidase (*GUS*) reporter gene expression observed by histochemical staining. **e**, Quantitative detection of the GUS enzyme in leaves inoculated with different recombinant vectors at different time points using a microplate plate-based GUS fluorescence assay. The error bars indicate the mean ± s.d.; *n* = 3 biological replicates. Significant differences were tested using a two-tailed *t*-test (***P < 0.0005). **f**, Phenotypic comparison of six accessions under heat treatment for 96 h. r1–r4: Tifleaf3, PI583800, PI526529 and PI587025 (HR group). s1 and s2: PI521612 and PI537069 (HS group). The HS plants were more wilted than the HR plants. **g**, Three examples showing the presence of fixed SVs in the HR groups (≥3 accessions) near three heat-related protein genes. Descriptive gene models are presented below the bar charts. The error bars indicate the mean ± s.d.; *n* = 3 biological replicates.

**Extended Data Fig. 9:**
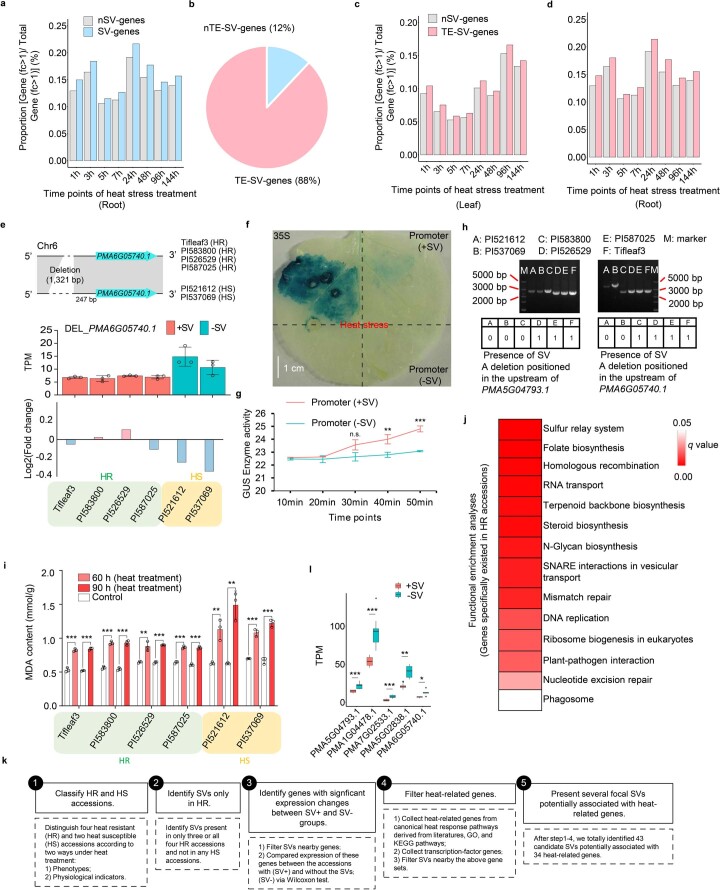
Potential contributions of structural variations (SVs) to heat tolerance in pearl millet. **a**, Proportion of genes with expression fold changes over 1 compared to total gene expression. nSV-genes and SV-genes: genes not located near SVs (over 5 kb) and genes located near SVs (within 5 kb), respectively. **b**, Composition of genes close to SVs overlapping with transposons (TE-SV-genes) or not overlapping with transposons (nTE-SV-genes). **c-d**, These two panels are similar to panel **a** but compares TE-SV-genes to nSV-genes. **e**, One example shows the presence of a fixed SV in the HR group near *PMA6G05740.1*. The lower panel shows the gene expression changes in *PMA6G05740.1* under 24 h heat treatment in the HR and HS groups. Error bars, mean ± s.d.; n = 3 biological replicates. TPM: transcripts per million. ‘+SV’ and ‘-SV’: accessions with and without SVs, respectively. **f-g**, Transformation of the *PMA6G05740.1* promoter in tobacco leaves. f: GUS phenotype observed by histochemical staining. Scale bar indicates 1 cm. g: Quantitative detection of GUS enzyme levels with a fluorescence microplate in leaves. Error bars, mean ± s.d.; n = 3 biological replicates. Significant differences were tested by two-tailed *t*-test (*P < 0.05, ***P < 0.0005; n.s., not significant). **h**, PCR validation of two SVs positioned in upstream regions of *PMA5G04793.1* and *PMA6G05740.1*. Each experiment is performed once. The images were cropped from the images in the Source data file. **i**, Malondialdehyde (MDA) levels of four heat-resistant (HR) and two heat-susceptible (HS) accessions. Error bars, mean ± s.d.; n = 3 biological replicates. Significant differences were tested by two-tailed *t*-test (**P < 0.01, ***P < 0.0005). **j**, Functional enrichment analyses of genes specifically found in the HR group. **k**, Pipeline of identifying focal candidate SVs potentially related to expression changes of nearby heat-related genes. **l**, Comparisons of the expression (TPM) of five genes between accessions with (+SV) and without SVs (-SV). These five genes are from panel e in this figure and from Fig. 5c and g. *PMA5G04793.1*, *PMA1G04478.1*, and *PMA7G02533.1*: n = 9 biological replicates for ‘+SV’ and ‘-SV’, respectively; *PMA5G02838.1* and *PMA6G05740.1*: n = 12 biological replicates for ‘+SV’ and n = 6 for ‘-SV’. Center line, median; box limits, upper and lower quartiles; whiskers, 1.5x interquartile range; and dots represent outliers. Significant differences were tested by two-tailed *t*-test (*P < 0.05, **P < 0.01, ***P < 0.0005). Source data are provided as a Source Data file. Source data

Previous reports revealed that SVs could affect the transcription of nearby genes^16,17,33^; our data showed that nearly half of SVs were near genes (Fig. 2d). Therefore, we investigated the influence of SVs on the expression of nearby genes that responded to heat stress. The results showed that SVs were enriched in nearby genes showing changes in gene expression in all accessions and that genes located near SVs are probably more responsive to heat stress (Fig. 5a,b, Extended Data Fig. 9a–d and Supplementary Note 7). We further validated two SVs that could cause transcriptional changes in nearby genes via a transient gene expression experiment in tobacco (*Nicotiana tabacum*) leaves and used PCR to confirm these two SVs (Fig. 5c–e, Extended Data Fig. 9e–h and Supplementary Note 8).

To identify potential SVs related to transcriptional changes of particular heat-related genes, we distinguished four HR (Tifleaf3, PI583800, PI526529 and PI587025) and two HS (PI521612 and PI537069) accessions based on the distinct phenotypes and physiological indicators of these accessions when grown under heat treatment (Fig. 5f, Extended Data Fig. 9i,j and Supplementary Note 6.2). Considering that different breeds in the same group may use different genes to respond to heat stress, we focused on 2,354 SVs present in only three or all four HR accessions and nearby 2,769 genes. We designed an analysis pipeline to screen out 44 candidate SVs potentially related to the expression changes of 34 heat-related genes (Extended Data Fig. 9k, Supplementary Table 13 and Supplementary Note 7). Almost all these genes (33 out of 34) were responsive to heat stress based on our RNA-seq data and 11 genes (32.35%) were included in ER-related gene pathways (Supplementary Table 14), suggesting potential contributions of the neighboring SVs to the HSR. Notably, we found four fixed SVs between the HR and HS groups in the vicinity of *PMA1G04478.1* and *PMA7G02533.1* encoding two HSP70 proteins (https://www.kegg.jp/entry/K03283) and *PMA5G02838.1* encoding one heat shock chaperonin-binding protein, which were associated with differences in gene expression in the HR group than those in the HS group (Fig. 5g and Extended Data Fig. 9l). Interestingly, *PMA1G04478.1* and *PMA5G02838.1* in the ER-related pathway were also identified and the main response of pearl millet to heat stress was found in this pathway (Fig. 5g and Supplementary Tables 13 and 14). In general, the transcription levels of these three genes, which have essential roles in the HSR, were probably affected by their nearby SVs, further demonstrating that these SVs might have important roles in the heat tolerance of pearl millet.

### Contributions of SVs to heat adaptation and domestication

**Fig. 6:**
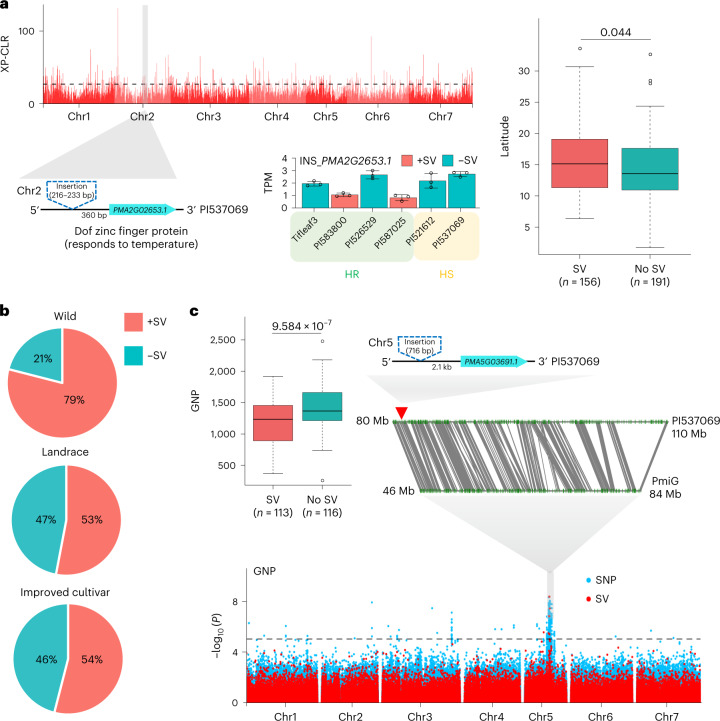
SVs contribute to heat tolerance adaptation and domestication. **a**, Upper left, results of an SV-based selection sweep analysis between two groups located in tropical and temperate zones. The black dashed line represents a cutoff window in which the top 1% data points were selected as sweep regions. Bottom left, the expression of *PMA2G02653.1* is influenced by a nearby SV. The error bars indicate the mean ± s.d.; *n* = 3 biological replicates. Right, comparison of the latitudinal distribution of the two SV-related haplotypes in pearl millet accessions. The center line indicates the median; the box limits indicate the upper and lower quartiles; the whiskers indicate 1.5 times the IQR; the dots represent the outliers. Significant differences were tested using a two-tailed *t*-test and are shown as *P* values. XP-CLR, cross-population composite likelihood ratio. **b**, Frequencies of SVs in wild and landrace accessions and improved cultivars. **c**, PAV–GWAS of SVs associated with the GNP trait based on the graph-based pan-genome. The center line indicates the median; the box limits indicate the upper and lower quartiles; the whiskers indicate 1.5 times the IQR; the dots represent the outliers. Significant differences were tested using a two-tailed *t*-test and are shown as *P* values. PmiG: Tift 23D2B1-P1-P5. The black dashed line represents the significance threshold based on a −log_10_(*P*) > 5.

**Extended Data Fig. 10:**
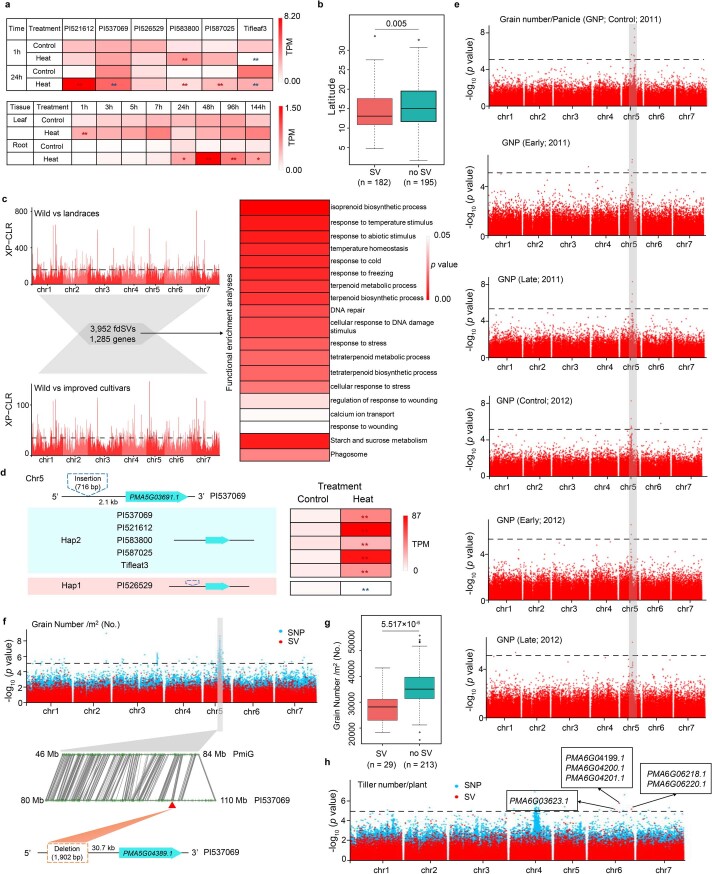
Contributions of structural variations (SVs) to heat tolerance adaptation and domestication. **a**, Expression patterns of *PMA2G02653.1*. Significant differences were performed by DESeq2 to determine fold change (FC) of gene expressions between control and heat-treatment groups (two-tailed Wald test). The FC was determined by * (0 < |log2FC|< 1, FDR-adjusted *P* < 0.05) or ** (|log2FC|>= 1, FDR-adjusted *P* < 0.05). The blue and red */** represent gene down- and upregulation, respectively. **b**, Comparison of the latitudinal distributions of the two SV-related haplotypes of pearl millet accessions. Center line, median; box limits, upper and lower quartiles; whiskers, 1.5x interquartile range; and dots represent outliers. Significant differences were tested by two-tailed *t*-test and shown by *p* value. **c**, SV-based selection sweep analyses. A total of 3,952 SVs with population frequency differences (fdSVs) were identified in both comparisons and harbored 1,285 genes. The black dotted line represents a cut-off window in which the top 1% of data points were selected as the sweep region. The right panel represents the functional enrichment analyses of the 1,285 genes. **d**, Expression levels of one candidate gene (*PMA5G03691.1*)-related haplotype. Significant differences were performed by DESeq2 to determine fold change (FC) of gene expressions between control and heat-treatment groups (two-tailed Wald test). The FC was determined by * (0 < |log2FC|< 1, FDR-adjusted *P* < 0.05) or ** (|log2FC|>= 1, FDR-adjusted *P* < 0.05). The blue and red ** represent gene down- and upregulation, respectively. **e**, PAV-GWAS of SVs associated with grain number/panicle (GNP). Control: condition without stress. Early: early drought stress inhibiting irrigation from one week before flowering until maturity. Late: late drought stress conducted during early grain-filling by inhibiting irrigation from 50% flowering time until maturity. The regions marked by grey box indicate the GNP-related QTL could be captured by different conditions. **f**, PAV-GWAS of SVs associated with the grain number/m^2^ (GNM2) trait. **g**, Comparison of GNM2 between accessions with SVs and without SVs. Center line, median; box limits, upper and lower quartiles; whiskers, 1.5x interquartile range; and dots represent outliers. Significant differences were tested by two-tailed *t*-test and shown by *p* value. **h**, PAV-GWAS of SVs associated with the tiller number/plant (Till) trait. PmiG: Tift 23D2B1-P1-P5. The dotted-line represents the significance threshold of -log10 (*p* value) > 5.

To characterize the SVs underlying heat tolerance during adaptation in a pearl millet population (SRP063925)^7^, we genotyped SVs by mapping all of the resequences to our graph-based pan-genome and identified a total of 124,532 SVs. We focused on the SVs with population frequency differences (fdSVs) between accessions from tropical and temperate zones by applying a sliding window methodology^34^ (Supplementary Note 7). In total, 1,471 genes were annotated against 269 selection sweep regions harboring 4,411 fdSVs (Fig. 6a). Interestingly, we found that 27 of these genes were significantly (*P* = 0.038; chi-squared test) and functionally annotated as belonging to ER-related pathways (that is, the Kyoto Encyclopedia of Genes and Genomes (KEGG) pathway ko04141) (Supplementary Tables 15 and 16). From the 591 genes whose expression was previously shown to be associated with SVs (Supplementary Table 13), we identified 25 genes near 27 fdSVs that were present only in the HR group; their expression levels were significantly correlated with the presence of fdSVs (Supplementary Table 17). Notably, one of the fdSVs was positioned close to (360 bp) and upstream of *PMA2G02653.1*, a gene encoding a protein in the zinc finger family that has a role in the ER system^35–37^. This gene was enriched in Gene Ontology (GO) terms associated with the response to temperature stress (GO: 0050826) and was also responsive to heat stress (Extended Data Fig. 10a). We further identified this fdSV as present in accessions that were preferentially located in higher-latitude regions (Fig. 6a and Supplementary Note 7). In general, these results revealed the contributions of SVs possibly associated with the ER system to heat stress adaptation.

To characterize the domestication of pearl millet with a shift toward higher heat tolerance, we used the above pearl millet population (SRP063925) to identify 113 selection sweep regions harboring 3,952 fdSVs overlapping with 1,285 genes between the landrace and improved cultivars relative to the wild accessions (Extended Data Fig. 10c and Supplementary Table 1). Functional enrichment analyses showed that these genes were associated mainly with stress-related GO terms, including temperature, abiotic stimulus and isoprenoid biosynthetic process (Extended Data Fig. 10c). We also found that 79.3% of those genes (1,019 out of 1,285) exhibited transcriptional changes (Supplementary Table 18), indicating that fdSVs potentially influence domestication genes under heat stress. In addition, 17 of these genes near 16 fdSVs were present only in the HR group and the fdSVs were significantly correlated with their gene expression levels; among these genes, *PMA2G02653.1* was also related to temperature adaptation (Fig. 6a).

Additionally, we found that a 716-bp insertion (SV) was present in a higher proportion of the wild accessions than the landrace ones and improved cultivars (Fig. 6b). This insertion was positioned 2.1 kb upstream of *PMA5G03691.1*, which encodes a coiled-coil 90B-like protein that is probably responsible for pollen germination and is associated with the grain number per panicle (GNP) trait. The presence of this insertion was possibly correlated with heat-induced gene expression (Extended Data Fig. 10d). We then conducted a genome-wide association study (GWAS) examining the associations of the 124,532 PAVs and 1,455,924 SNPs with GNP in a population reported by Varshney et al.^7^ (Supplementary Table 19). An association peak on chromosome 5 showed an overlap between PAVs and SNPs. This quantitative trait locus corresponds to grain number^7^. In our study, we found *PMA5G03691.1* and an insertion in the close vicinity of this quantitative trait locus (Fig. 6c, Supplementary Table 19 and Supplementary Note 7). We next observed this insertion in 113 accessions with lower GNP values than 116 accessions without the SV (Fig. 6c). These results suggested that this insertion was probably under positive selection during domestication and influenced the responsiveness of nearby genes to heat, possibly contributing to seed production in pearl millet grown at higher temperatures. Furthermore, we identified a total of 142 PAVs that were each associated with one or more traits (20 traits in total), which might provide insights into the contributions of these SVs to pearl millet molecular breeding (Supplementary Table 19 and Supplementary Note 7). Collectively, these results demonstrate the utility of pearl millet graph-based pan-genome analysis for the identification of both heat tolerance adaptation and its relationship to domestication.

### Resistance to heat in pearl millet depends on the ER system

**Fig. 7:**
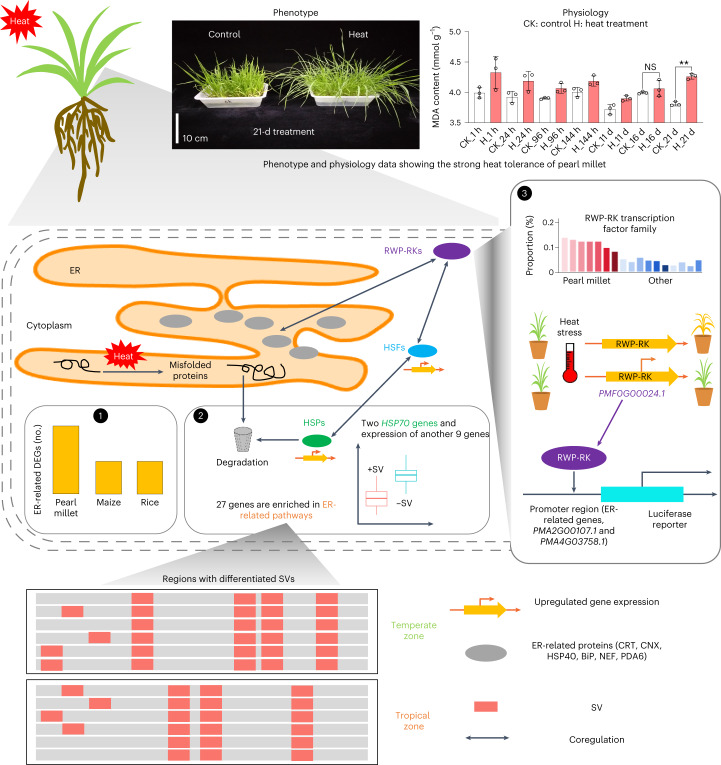
A proposed mechanism by which heat tolerance is integrally related to the transport system of the ER. After heat stress (H) for 21 d, only a small proportion of leaves exhibited wilting relative to the control (CK) group; leaves showed physiological changes (MDA) for up to 21 d, suggesting that pearl millet exhibits strong heat resistance. Significant differences were tested using a two-tailed *t*-test (**P < 0.01). We leveraged multi-omics analyses to reveal a possible mechanism of heat tolerance in the ER transport system: (1) compared with maize and rice, pearl millet showed a higher proportion of ER-related genes that were differentially expressed, indicating a quicker response to heat stress in this system; (2) this heat stress led to the production of misfolded proteins that could be recognized and degraded via the cooperation of ER-related proteins such as CRT, CNX, BiP, NEF, PDA6 and HSP. *HSF* genes might be involved in the heat response because their expression is upregulated and they can coregulate *HSP* genes^45,46^. SVs surrounding 11 ER-related genes probably contributed to this response. For instance, one SV was associated with the expression of an ER-related gene, *HSP70*, which plays a role in the degradation of misfolded proteins. Additionally, 27 genes enriched in the ER system were located in regions with differentiated SV distributions between two populations in temperate and tropical zones; (3) furthermore, an *RWP-RK* gene (*PMF0G00024.1*) from an expanded transcription factor family was confirmed as a positive regulator involved in heat resistance and was coregulated by ER-related and *HSF* genes. We finally used dual luciferase assays to confirm that this *PMF0G00024.1* gene could transactivate genes encoding ER-related BiP (*PMA2G00107.1*) and OST (*PMA4G03758.1*).

We performed integrated multi-omics analyses supplemented with *cis*-genetic functional verification to propose a possible mechanism by which the superior heat tolerance of pearl millet is related to the expansion and altered expression of genes involved in the ER system (Fig. 7). In particular, the ER system showed a quicker response to high temperature in pearl millet than in maize and rice. Abundant evidence has shown that SVs participate in the heat tolerance response by affecting gene regulation; for example, SVs between HR and HS materials led to differential expression levels of 11 ER-related genes. Several other distinctly differentiated SVs in ER-related genes were also associated with the heat stress adaptation of pearl millet populations at different temperatures. Moreover, by means of functional analysis, we confirmed that one gene (*PMF0G00024.1*) from an expanded RWP-RK transcription factor family acted as a positive regulator of heat resistance; this transcription factor also transactivated one ER-related gene. These observations indicate that SVs and *RWP-RK* genes may coregulate the quick response to heat stress with ER-related genes in pearl millet.

## Discussion

Pearl millet is an ideal model for investigating the mechanisms underlying plant heat resistance^9^. We identified distinctly differentiated SVs in ER-related genes that were associated with the heat stress adaptation of pearl millet populations at different temperatures (Fig. 6a); however, we did not find genes in SNP-based selection sweep regions that showed significant enrichment in ER-related pathways. These findings indicate that SV-based population analyses can capture genetic variations complementary to SNPs, providing additional information about the diversity losses caused by population bottlenecks during plant adaptation^38^. In addition, the expansion of RWP-RK transcription factors was likely related to LTR and these factors coregulated heat tolerance with ER-related and heat stress-related genes (Figs. 3 and 4). RWP-RK transcription factors have an important role in the nitrogen starvation response and gametophyte development in plants^39,40^. However, no heat tolerance-related functions of these transcription factors have been reported. Our findings expand the possible functions of RWP-RK transcription factors and illustrate a possible diversification in which this family of transcription factors is responsible for multiple stress condition responses in plants. This finding supports a previous hypothesis that pearl millet probably includes abundant heat tolerance-related genetic resources^6^.

The graph-based pan-genome resource offers several potential tools to improve the breeding process in pearl millet. We developed a comprehensive SV map of pearl millet to identify signals associated with phenotypes (that is, GNP) (Fig. 6c), which enables us to investigate potential mechanisms influencing nearby genes that are challenging to detect based only on SNP genotyping. This pan-genome also provides a new window for identifying evolutionary processes, such as the formation of adaptative SVs, to elucidate demographic and selection processes in pearl millet. The dispensable genome within the pan-genome resource offers a pathway for identifying genes associated with traits such as abiotic stress resistance or production, which would benefit the selection of suitable materials for use as breeding targets in pearl millet. In our pan-genome, PmiG showed a higher ratio of private gene families relative to the other assemblies (Fig. 1c), possibly caused by the relatively fragmented sequences generated by previous short-read sequencing or assembly techniques^7,41–43^. A similar result was reported in a soybean pan-genome study^17^. The relatively lower contig N50 value intuitively suggested that the PmiG genome sequence is more fragmented (Table 1), which would lead to a lower average length of genes and coding sequences and a higher proportion of short genes (<1 kb) (Extended Data Fig. 4c,d). Thus, fragmentation of assembled sequences would result in incomplete prediction of genes, potentially contributing to the private gene set in the PmiG. Nonetheless, the PmiG, as the first published pearl millet genome, has been widely used as a reference genome in the pearl millet community^20,25,44^. Including it in our pan-genome research will help to refer to the basis of previous research and provide a smooth transition to the era of high-quality pearl millet genome research.

In conclusion, our study uses a pan-genome approach coupled with multi-omics to comprehensively investigate plant response mechanisms to heat stress. This work provides an excellent reference for future studies on stress tolerance, especially in non-model plants. Our study also offers an approach for breeding crop varieties with enhanced tolerance to various stresses that can cope with the diverse challenges imposed by the changing climate.

## Methods

### Sampling and sequencing

Ten pearl millet accessions (PI537069, PI521612, PI526529, PI587025, PI583800, PI343841, PI186338, PI250656, PI527388 and Tifleaf3) were obtained as representative plants from different geographical regions. All ten accessions were planted in a greenhouse at a density of three plants per pot (filled with nutrient soil), including nine plants of each accession, and grown at a temperature of 26 °C during the light period (14 h of light) and 22 °C during the dark period (10 h of darkness). Thirteen-week-old leaves were collected and immediately frozen in liquid nitrogen for the extraction of genomic DNA using a DNAsecure Plant Kit (TIANGEN). Library construction and Illumina, Hi-C, PacBio and Bionano sequencing were performed at Novogene (Supplementary Note 1).

### Genome survey

The genome size of pearl millet was estimated using *k*-mer frequency analysis based on the Lander–Waterman algorithm^47^. We divided the total length of sequence reads by the sequencing depth represented by the peak value of the frequency curve. The following formula was used to estimate genome size: (*N* × (*L* − *k* + 1) − *B*)/*D* = *G*, where *N* is the total number of sequence reads, *L* is the average length of the sequence reads, *k* is the *k*-mer length (17 bp), *B* is the total number of low-frequency *k*-mers (frequency ≤ 1 in this analysis), *G* is the genome size and *D* is the overall estimated depth based on the *k*-mer distribution^48^. Additionally, flow cytometry was used to confirm the estimated genome size according to a reported method^49^ with a BD FACSCalibur flow cytometer and the fluorochrome propidium iodide.

### Initial assembly

The PacBio HiFi reads were used to assemble the initial contigs in the Hifiasm (v.0.13-r308)^50^ package with default parameters. The Pruge_haplotig (v.1.1.0)^51^ tool was used to process genomic heterozygous regions to remove redundancy in the genomes using default parameters with several exceptions: -a 50.

### Scaffolding with Bionano optical maps

The filtered raw DNA molecules in BNX format were aligned, clustered and assembled into a Bionano optical map using the Bionano Genomics assembly pipeline. Then, a BNX file recorded the basic labeling and DNA length information was converted with the AutoDetect in Bionano Solve package (v3.5.1) (https://bionanogenomics.com/support/software-downloads/). The initial assemblies were aligned to the Bionano data and then analyzed with RefAligner in Bionano Solve package (v3.5.1). The alignments were visualized with a snapshot in IrysView in Bionano Solve package (v3.5.1). Finally, genome maps were combined with the initial assembly to produce hybrid scaffold genome maps using the Bionano Solve package (v.3.5.1) with the parameters -B 1 -N 1.

### Pseudochromosome construction

Linkage information for the scaffold and initial assembly was obtained by aligning high-quality Hi-C data to the preceding assemblies using the Burrows–Wheeler Aligner (BWA) software (v.0.7.8)^52^. Chromosome-scale scaffolds were anchored based on linkage information, restriction enzyme sites and the string graph formulation using the ALLHIC (v.0.9.8)^53^ package with the following parameters: -K 7 -minREs 50--maxlinkdensity 3--NonInformativeRatio 0. Placement and orientation errors showing obvious discrete chromatin interaction patterns were adjusted manually. For those accessions without Hi-C data, we used collinearity with the PI537069 assembly for clustering and orientation to generate chromosome-level assemblies.

### Genome assessment

To evaluate the assembly quality of the genomes, BUSCO (v.4.1.2; http://busco.ezlab.org/)^54^ and the CEGMA (v.2.5) (http://korflab.ucdavis.edu/dataseda/cegma/)^55^ were used to check the completeness of the genome assembly or annotation. The draft assemblies were further evaluated by mapping the high-quality Illumina paired-end reads to the genome assembly using the BWA–MEM (v.0.7.8)^52^ algorithm. The quality of the genome assemblies was further evaluated using LTR TE completeness based on the LAI tool wrapped in LTR_retriever (v.2.8)^21^ and using Merqury (v.1.3)^22^ with the default parameters.

### Annotation of repetitive sequences

Transposons were annotated by combining two strategies, that is, homolog and de novo predictions. For the homology-based approach, the Repbase TE library^56^ and the TE protein database (http://www.repeatmasker.org/cgi-bin/RepeatProteinMaskRequest) were used to mask TEs with the RepeatMasker (v.4.0.5)^57^ and RepeatProteinMask (v.4.0.5)^57^ tools. Under the de novo-based method, LTR_FINDER (v.1.0.7) (https://github.com/xzhub/LTR_Finder)^58^, PILER (v.1.0) (https://www.drive5.com/piler/)^59^, RepeatScout (v.1.0.5) (https://github.com/mmcco/RepeatScout)^60^ and RepeatModeler (v.1.0.8) (http://www.repeatmasker.org/RepeatModeler.html)^61^ were used to build a de novo repeat library. This new library was used to mask TEs with the RepeatMasker tool^57^. We estimated the insertion times of the intact LTR retrotransposons. Sequences from the 5′ and 3′ LTRs were aligned with MUSCLE^62^ (v.3.8.31). Nucleotide variations (*λ*) in the 5′ and 3′ ends of intact LTR retrotransposons were calculated and DNA substitution rates (*K*) were calculated using *K* = −0.75ln (1 – 4*λ*/3). The insertion time of these LTR retrotransposons was estimated based on *T* = *K*/2*r*, where *r* is 1.3 × 10^−^^8^ per site and per year^63^.

### Annotation of gene structure

Gene annotation was conducted by combining de novo-, homolog- and transcriptome-based predictions. For the homolog-based approach, we downloaded homologous proteins from the *A. thaliana*, *Z. mays*, *S. bicolor*, *O. sativa*, *S. italica* and pearl millet genomes (Phytozome 13, https://phytozome.jgi.doe.gov/pz/portal.html; NCBI, https://www.ncbi.nlm.nih.gov/) and aligned them to the pearl millet genome with Tblastn (v.2.2.26)^64^ using an expected value of 1 × 10^−5^. Solar (v.0.9.6)^65^ was used to combine the BLAST hits (Homo‐set), which were used to predict the exact gene structures of the corresponding genomic regions with GeneWise (v.2.4.1)^66^ (https://www.ebi.ac.uk/Tools/psa/genewise). For the transcriptome-based approach, RNA-seq data from Illumina were mapped to the assembled genome with TopHat (v.2.0.13)^67^, followed by Cufflinks (v.2.1.1)^68^. In addition, Trinity (v.2.1.1)^69^ was used to assemble the RNA-seq data and its output was used to create pseudo-expressed sequence tags, which were then mapped to the assembly. Gene models were predicted by using the Program to Assemble Spliced Alignments (PASA) genome annotation tool^70^. This gene set was denoted as the PASA-T-set and was used to train ab initio gene prediction programs. For the de novo-based approach, five ab initio gene prediction programs, including AUGUSTUS (v.3.2.3) (http://augustus.gobics.de/)^71^, GENSCAN (v.1.0) (http://genes.mit.edu/GENSCAN.html)^72^, GlimmerHMM (v.3.0.1) (http://ccb.jhu.edu/software/glimmerhmm/)^73^, geneid (v.1.4) (http://genome.crg.es/software/geneid/)^74^ and SNAP (v.2013.11.29) (http://korflab.ucdavis.edu/software.html)^75^ were used to predict coding regions from the repeat-masked genome. Finally, EVidenceModeler (v.1.1.1)^76^ was used to combine all gene model evidence obtained from these three strategies.

### Functional annotation of protein-coding genes

Two protein sequence databases, Swiss-Prot (http://web.expasy.org/docs/swiss-prot_guideline.html) and the NR Protein Sequence database (ftp://ftp.ncbi.nih.gov/blast/db/) were used to annotate protein-coding genes. Protein domains were predicted using InterProScan (v.4.8) and HMMER (v.3.1) (http://www.hmmer.org/) based on the InterPro (v.32.0) (http://www.ebi.ac.uk/interpro/) and Pfam (v.27.0) (https://pfam-legacy.xfam.org/) databases, respectively^77–80^. These two databases provide a portal for obtaining GO terms (http://geneontology.org/http://www.geneontology.org/page/go-database)^81^. The pathways of the genes were identified via BLAST searches against the KEGG database (v.53) (http://www.kegg.jp/kegg/kegg1.html)^82^ with an expected value cutoff of 1 × 10^−5^.

### Pan-genome construction

We constructed a pan-genome using the 11 pearl millet assemblies. The core and dispensable gene sets among the 11 pearl millet genomes were estimated based on gene family clustering using OrthoFinder (v.2.3.1)^83^. All protein sequences were subjected to homologous searches using BLASTP with an expected value of 1 × 10^−5^. Protein sequences were clustered into paralogous and orthologous sequences using OrthoFinder with an inflation parameter of 1.5.

### SV identification

To build a genetic variance atlas for the 11 pearl millet genomes, we aligned the other ten genomes to the PI537069 reference genome using MUMmer (v.4.0.0)^84^. The alignment of the genomes was performed using NUCmer^84^ (--c 1000--maxgap=500) and the alignment block filter was implemented using a delta filter in one-to-one alignment mode (−1). Blocks longer than 1,000 bp were used for further analysis. We used the SV function of the MUMmer (SVMU) pipeline to automate PAV discovery by parsing the results of NUCmer. From the SVMU results, SV-based insertions or deletions (with the tag INS or DEL) were treated as PAVs and CNVs were treated as CNVs. Inversion events (referring to SVs more than 1 kb in length) were identified by SVMU. SyRI (v.1.6.3) (https://github.com/schneebergerlab/syri)^85^ was used to identify translocation regions. We also used PI537069 as a reference to construct a graph-based genome with the vg tool (v.1.25.0) (https://github.com/vgteam/vg)^86^. To genotype the population SVs, the Illumina short reads (SRP063925) of each accession were mapped to the graph-based genome using the vg tool with default parameters.

### Transcription factor family identification and analysis

To identify and compare transcription factor families in pearl millet and other species, we collected the protein sequences of *A. thaliana* (TAIR10)^87^, *Z. mays* (B73_RefGen_v4)^88^*, B. distachyon* (v.3.1) (https://phytozome-next.jgi.doe.gov/info/Bdistachyon_v3_1)^89^, *O. thomaeum* (v.1.0)^90^, *P. hallii* (PHallii_v3.1)^91^, *D. oligosanthes* (ASM163321v2)^92^, *O. sativa* (IRGSP-1.0)^93^, *S. bicolor* (Sorghum_bicolor_NCBIv3)^94^, *H. vulgare* (Hvulgare_462_r1)^95^, *S. italica* (Setaria_italica_v2.0)^96^, *S officinarum* (v.1.0)^97^, *M. esculenta* (v.1.0)^98^, *C. annuum* (v.1.6)^99^, *P. miliaceum* (v.2.0)^100^, *E. coracana* (v.2.0)^101^, *D. exilis* (DiExil)^102^ and *S. viridis* (v.2.0)^103^. The iTAK tool (v.1.7a)^104^ was used for transcription factor prediction with default parameters. To avoid bias caused by differences in the number of genes among the different plants^105^, we calculated the proportion of transcription factor as *N*_TF_/*N*_total_, where *N*_TF_ is the number of transcription factors and *N*_total_ is the total number of genes in the corresponding plant. Moreover, we predicted the binding sites of RWP-RK transcription factors with the FIMO tool (v.5.3.2) (https://meme-suite.org/meme/meme_5.3.2/doc/fimo.html)^106^.

### Contributions of SVs to nearby gene expression

To investigate whether the SVs could broadly influence nearby gene expression, we used RNA-seq dataset B for the six accessions subjected to 1 h of control conditions (Supplementary Table 3). The SVs were divided into 11 categories: deletion of coding DNA sequence start (DELCDSstart); deletion overlapping the 5-kb downstream region (DELdown); deletion of exons (DELexons); deletion of the whole gene (DELgene); deletion overlapping the 5-kb upstream region (DELup); duplication (Dup); insertion in the 5-kb downstream region (INSdown); insertion in exons (INSexons); insertion in introns (INSintrons); insertion in the 5-kb upstream region (INSup); and the presence of SVs (PresenceSVs).

### PAV–GWAS

To explore the usefulness of the graph-based genome and identify SV-driven alterations of genes controlling important agronomic traits, we conducted a PAV–GWAS analysis. After PAV filtration (removal of PAVs with a minor allele frequency < 0.05 or missing rate > 0.1), a total of 124,532 PAVs were used to perform PAV–GWAS in 242 accessions. Association analysis was conducted using the GEMMA (v.0.94.1) software package^107^. For the mixed linear model analysis, we used the equation *y* = *Xα* + *Sβ* + *Kµ* + *e*, where *y* represents the phenotype, *X* represents the genotype, *S* is the structure matrix and *K* is the relative kinship matrix. *Xα* and *Sβ* represent fixed effects and *Kμ* and *e* represent random effects. The top three principal components were used to build the *S* matrix for population structure correction. The matrix of simple matching coefficients was used to build the *K* matrix.

### Determination of physiological indicators

Seeds (2.00 g) of Tifleaf3 were cultured in a plastic box (10 × 15 × 6 cm) under growth conditions of 14 h light at 26 °C and 10 h darkness at 22 °C. The 13-day-old seedlings (V3 stage: third leaf visible at the vegetative stage) were divided into three groups: a high-temperature treatment group (45 °C under light for 14 h and 40 °C in darkness for 10 h), a heat treatment group (40 °C under light for 14 h and 35 °C in darkness for 10 h) and a control group (26 °C under light for 14 h and 22 °C in darkness for 10 h). After 1, 24, 96 and 144 h, and 11, 16, 21, 26, 31, 36 and 41 d of heat treatment or control conditions, leaves were subjected to the measurement of relative water content, relative conductivity and MDA content. In addition, the materials (PI537069, PI521612, PI526529, PI587025, PI583800 and Tifleaf3) used for pan-genome sequencing were cultured under the same conditions described above and divided into a high-temperature treatment group and a control group. After treatment for 1, 24, 60 and 96 h, leaves were collected for the determination of relative water content, electrical conductivity and MDA content. Transgenic rice and WT rice were cultured at 26 °C under light for 14 h and 22 °C in darkness for 10 h each day for 45 d and then divided into two groups: a high-temperature treatment group (45 °C under light for 14 h and 45 °C in darkness for 10 h) and a control group (26 °C under light for 14 h and 22 °C in darkness for 10 h). MDA content and POD and SOD enzyme activities were quantified in the plants after 12 h and 72 h of heat treatment.

### Measurement of POD, MDA and REC

Leaves (0.1 g) were ground and 1.5 ml of PBS solution (150 mM) was added. The mixtures were centrifuged at 12,879.36*g* for 20 min at 4 °C. The supernatant was then collected. For the determination of MDA activity, 0.5 ml of enzyme extract was added to 1 ml of reaction solution (20% trichloroacetic acid and 0.5% thiobarbituric acid) and the mixture was incubated in a 95 °C water bath for 30 min. Thereafter, the mixture was placed in an ice bath at room temperature (25 °C) and centrifuged at 12,879.36*g* for 10 min. The absorbance was recorded at 532 nm and 600 nm using a spectrophotometer (Sorvall ST 16). For the determination of POD activity, a 1.5 ml reaction system was used. First, 925 μl sodium acetate (100 mM) was added, after which 0.5 ml guaiacol (0.25%) and 25 μl enzyme extract were added. After mixing, 50 μl of hydrogen peroxide (0.75%) was added to the mixture. The absorbance was recorded at 470 nm every 10 s. SOD enzymatic activity was determined as described by Dhindsa et al.^108^. Starting with 50 μl of crude enzyme solution, 1.1 ml of 50 mM phosphate buffer, 100 μl of 0.06 mM riboflavin, 100 μl of 195 mM l-methionine, 50 μl of 0.003 mM EDTA and 100 μl of 1.125 mM nitroblue tetrazolium were added. In addition, two tubes without enzyme extract were included as controls. The reaction was performed under 3000 lx light for 30 min and the reaction was terminated in the dark. Absorbance was recorded at 560 nm. For the measurement of REC, 0.1 g samples of fresh leaves were collected with six biological replicates. The leaves were wrapped using gauze and placed in a 50-ml Eppendorf tube and 20 ml of pure water was added to completely cover the leaves. The tube was placed in an incubator at room temperature (25 °C). After 25 h, the S1 EC was measured and the sample was kept in a boiling water bath for 30 min. The S2 EC was measured when the water had cooled to room temperature (25 °C). The REC was calculated using the following equation: REC = S1/S2 × 100%.

### Transcriptomic analyses of pearl millet under high temperature

Seeds (2.00 g) of six accessions of pearl millet were cultivated in a 10 × 15 × 6 cm plastic basin filled with quartz sand and placed in a growth chamber (26 °C under light for 14 h and 22 °C in darkness for 10 h). The culture conditions were as described by Sun et al.^109^. The V3 stage seedlings were equally divided into two groups: a high-temperature treatment group and a control (CK) group. The conditions of the high-temperature treatment group were 14 h under light at 45 °C and 10 h in darkness at 40 °C, while the CK group was cultured under unchanged conditions (26 °C and 22 °C). After 1 and 24 h of treatment, leaves were collected and stored at −80 °C. In addition, the seeds (2.00 g) of Tifleaf3 were grown under similar conditions and seedlings were divided into treatment and control groups as described above. The culture conditions of the heat-treated group were 14 h under light at 40 °C and 10 h in darkness at 35 °C; the control group was kept under unchanged conditions (26 °C and 22 °C). After treatment for 1, 3, 5, 7, 24, 48, 96 and 144 h, the roots and leaves of the seedlings were collected and stored at −80 °C. A total of 168 samples were collected and three biological replicates were set for each treatment and control. Each replicate consisted of the mixed tissues of 16 seedlings. To obtain the materials used for the annotation of gene structure, the ten accessions were planted in a greenhouse, with nine plants of each accession (26 °C under light for 14 h and 22 °C in darkness for 10 h). We collected leaves (three biological replicates), stems (one sample) and roots (one sample) 5 weeks after the planting of each accession to build 30 RNA-seq libraries. A Total RNA Kit (QIAGEN) was used to extract RNA from these samples to build a complementary DNA library (NEBNext Ultra Directional RNA Library Prep Kit for Illumina) in preparation for RNA-seq. After sequencing, the raw data were filtered with FastQC (v.0.11.9) (http://www.bioinformatics.babraham.ac.uk/projects/fastqc/)^110^. Transcripts were quantified with the Kallisto (v.0.46.2)^111^ software using PI537069 as a reference. Finally, DEGs (|log_2_(group 1/group 2)| ≥ 1, *P*_adj_ < 0.05) were identified with DESeq2 (v.1.26.0)^112^. GO and KEGG enrichment analyses were performed using the OmicShare tools (http://omicshare.com/tools) (*P* < 0.05). Moreover, for the processing of published maize and rice transcriptomic data, we downloaded raw reads from maize in the V3 stage under 38 °C (14 h under light and 10 h in darkness) stress and normal conditions (25 °C; 14 h under light and 10 h in darkness)^31^ and raw data from rice in the V3 stage grown under either 45 °C (13 h under light and 11 h in darkness) stress or normal culture condition (25 °C; 13 h under light and 11 h in darkness)^32^. The same methods and parameters were applied to the RNA-seq analysis of published maize and rice data.

### Transgenic plant validation

The *PMF0G00024.1* gene sequence was synthesized via synthetic gene sequence generation and was introduced to the pBWA (V)HS-CCDB vector under the control of the 35S promoter. Three hundred rice seeds without mildew spots that showed normal buds were sterilized with 75% alcohol for 1 min, soaked in sodium hypochlorite for 20 min, washed with sterile water three times and then placed into a culture medium to culture calluses. The culture was conducted under light at 26 °C for 20 d. In addition, a single *Agrobacterium* colony was cultured in medium in a shake flask to obtain an *Agrobacterium* resuspension with an OD_600_ of 0.2. The calluses were added to the *Agrobacterium* suspension step. After 10–15 min of infection, calluses were picked, placed in a cocultivation medium and incubated at 20 °C for 48–72 h. Subsequently, cultured calluses were transferred to a selection medium containing hygromycin and cultured for 20–30 d (26 °C in darkness) for the first selection. After the first selection, 180 calluses were transferred to a new culture medium and cultured for 7–10 d (26 °C in darkness) for the second selection step. Ninety callus tissues were obtained and differentiation and rooting were induced. Finally, a total of 20 seedlings were obtained. The resistant calluses were differentiated into seedlings and PCR detection was performed using the primers listed in Supplementary Table 20. The PCR-positive seedlings were transplanted into the soil (26 °C under light for 14 h and 22 °C in darkness for 10 h). When they reached the four-leaf stage, quantitative PCR with reverse transcription was performed with the primers *RWP1* and *RWP2*, with three technical repeats for each sample (Supplementary Table 20).

### Dual luciferase assays to assess the interaction between *RWP-RK* and ER-related genes

The open reading frames of *RWP-RK* (*PMF0G00024.1*) were inserted into the pGreenII62-SK vector to generate effector plasmids. The promoter sequence of *PMA2G00107.1* was synthesized by Hzykang and then cloned into the pGreenII 0800-LUC vector to generate reporter plasmids. Effector and reporter plasmids were expressed in tobacco leaves, mediated by *Agrobacterium* injection. Tobacco leaves in the injection area were collected and fluorescence activity was measured using a luciferase assay kit (cat. no. DL101, Vazyme Biotech). The primers used in this section are shown in Supplementary Table 20.

### Tobacco leaf transformation assays to assess the impact of SVs on gene expression

The promoter sequences were cloned into the T vector using the 5 min TA/Blunt-Zero Cloning Kit (cat. no.C601, Vazyme Biotech). We used PCR (enzyme mix, cat. no. P520, Vazyme Biotech) to add the vector sequence at the end of the promoter fragment and obtained the PBI121-GUS linearized vector (Supplementary Table 20). Circularization was performed according to the instructions of the Clone Kit (cat no. MC40101, Monad). The recombinant vectors were injected into *Nicotiana benthamiana* leaf cells using an *Agrobacterium*-mediated transfection system (GV3101). GV3101-pBI121-35s-GUS, GV3101-pBI121-Promoter-GUS and GV3101-pBI121-Promoter_SV-GUS were cultured to an OD_600_ of 0.6 before injection. Two hundred microliters of liquid from each treatment was infiltrated into the tobacco leaves. Gloves were changed after the infiltration of each construct to prevent contamination. Tobacco was pretreated at a high temperature for 24 h (40 °C for 8 h and 35 °C for 16 h) and then cultured under the same conditions for 2 d after injection. The blank group was cultured at 25 °C (8 h under light and 16 h in darkness) and sampled by injection. The histochemical staining and quantitative analysis of GUS in three independent biological replicates were performed as described by Jefferson et al.^113^.

### PCR validation of SVs

Genomic DNA was extracted from fresh leaves using a DP360 kit (TIANGEN) and PCR was performed using 2× Phanta Flash Master Mix (cat. no. P520, Vazyme Biotech). Five SVs were analyzed by PCR genotyping (condition: followed by 35 cycles of denaturation at 98 °C for 10 s, annealing at 60 °C for 5 s and extension at 72 °C for 5 s kb^−1^) using the primers indicated in Supplementary Table 20.

### Reporting summary

Further information on research design is available in the Nature Portfolio Reporting Summary linked to this article.

## Online content

Any methods, additional references, Nature Portfolio reporting summaries, source data, extended data, supplementary information, acknowledgements, peer review information; details of author contributions and competing interests; and statements of data and code availability are available at 10.1038/s41588-023-01302-4.

## Supplementary information

Supplementary Information Supplementary Figs. 1–3 and Note, including 8 sections.

Reporting Summary

Supplementary Tables Supplementary Table 1: Summary of 378 pearl millet accessions from the SRP063925 dataset and 16 accessions from our study. Supplementary Table 2: Summary of the ten pearl millet accessions for de novo assembly. Supplementary Table 3**:** Overview of the RNA-seq results of 168 samples. Supplementary Table 4: The log_2_ (heat/control) expression fold change of genes related to two stress-related pathways under eight time points (h) in leaf (L) and root (R) tissues of Tifleaf3 accession. Supplementary Table 5: Summary of genetic variations generated by comparing ten genomes to PI537069. Supplementary Table 6: Comparisons of SV predictions from SVMU and SyRI, Assemblytics, and smartie-sv tools. Supplementary Table 7: Validation of SVs in a pearl millet population. Supplementary Table 8: Validation of SVs in all de novo assembled genomes. Supplementary Table 9: Information of species used for comparing the proportion of *RWP-RK* among all genes (Fig. 3b) and used to build the RWP-RK TF phylogenetic tree (Supplementary Fig. 3). Supplementary Table 10: IDs of specific (clade A) and nonspecific (clade B) *RWP-RK* genes shown in a phylogenetic tree in Supplementary Fig. 3. Supplementary Table 11: Correlation analyses of ten *RWP-RK* genes to other genes. Significant differences were tested by two-tailed Pearson correlation test and shown by *P* value. Supplementary Table 12: The log_2_ (heat/control) expression fold change of ER-related genes under eight time points (h) in leaf (L) and root (R) tissues of Tifleaf3 and in six accessions of pearl millet after high temperature stress for 24 h. Supplementary Table 13: Summary of candidate SVs associated with expression of nearby genes. Significant differences were tested by two-tailed Wilcoxon test and shown by *P* values; they are corrected to be FDR-adjusted *P* values (*q* values). Supplementary Table 14: log_2_ (heat/control) expression fold change of 34 genes potentially associated with nearby SVs under eight time points (h) in leaf (L) and root (R) tissues. Supplementary Table 15: KEGG enrichment analyses of genes under 269 selection sweep regions. Significant differences were tested by two-tailed chi-squared test and shown by *P* values; they are corrected to be FDR-adjusted *P* values (*q* values). Supplementary Table 16: Annotation of 27 genes of ER-related pathways under selection sweep regions. Supplementary Table 17: The selective sweep regions covered 25 genes nearby 27 fdSVs only existing in the HR group. Significant differences were tested by two-tailed Wilcoxon test and shown by *p* values that are corrected to be FDR-adjusted *p* values (*q* values). Supplementary Table 18: The log_2_ (heat/control) expression fold change of genes under selective sweep regions under eight time points (h) in leaf (L) and root (R) tissues. Supplementary Table 19: Summary of PAVs associated with 20 traits identified by PAV–GWAS. The *P* values were calculated by GEMMA using a mixed linear model. Supplementary Table 20: Primer design.

## Data Availability

The raw sequencing data and transcriptome data of PI186338, PI250656, PI343841, PI521612, PI526529, PI527388, PI537069, PI583800, PI587025 and Tifleaf3 have been deposited in the NCBI Sequence Read Archive under BioProject accession no. PRJNA749489, PRJNA689619 and PRJNA756390. The assemblies of ten pearl millet have been deposited in NCBI GenBank under the accession no. JAMZRY000000000 (PI343841), JAMOAQ000000000 (PI250656), JAMKQL000000000 (PI186338), JAMKQK000000000 (PI527388), JAJHQD000000000 (PI587025), JAIFIR000000000 (PI537069), JAINUP000000000 (Tifleaf3), JAINUO000000000 (PI583800), JAINUN000000000 (PI526529) and JAINUM000000000 (PI521612). These assemblies are also available at http://117.78.45.2:91/download. The raw genome assembly data are available under accession no. PRJNA749489. The transcriptomic data are available under accession nos. PRJNA749489, PRJNA689619 and PRJNA756390. The public RNA-seq data used were downloaded from the NCBI and the BioProject accession no. is PRJNA520822. The public resequencing data used were downloaded from the NCBI and the accession no. is SRP063925. Source data are provided with this paper.

## Source data

Source Data Extended Data Fig. 3 Source data from the flow cytometry analysis.
Source Data Extended Data Fig. 5 Unprocessed gels.
Source Data Extended Data Fig. 9 Unprocessed gels.

## Code availability

All the analysis tools used in this study have been published before as described in the Methods and Reporting Summary.
