# Supplementary material for: Pangenomic analysis identifies structural variation associated with heat tolerance in pearl millet
Source: Nat Genet. 2023 Mar 2;55(3):507–18. doi: 10.1038/s41588-023-01302-4 (PMC10011142; doi:10.1038/s41588-023-01302-4)
Supplement: Supplementary file 1 — Supplementary Figs. 1–3 and Note, including 8 sections. [file 41588_2023_1302_MOESM1_ESM.pdf]

nature genetics

Article

https://doi.org/10.1038/s41588-023-01302-4

Pangenomic analysis identifies structural variation associated with heat tolerance in pearl millet

In the format provided by the authors and unedited

SUPPLEMENTARY INFORMATION

Contents

Supplementary Figures

1. Supplementary Fig. 1……………………………………………………………………2
2. Supplementary Fig. 2……………………………………………………………………3
3. Supplementary Fig. 3……………………………………………………………………4

Supplementary Note

1. Sequencing, assembly, and annotation of ten pearl millet genomes………………………….…5
2. Analysis of core, dispensable, and private genes…………………………………….……..…7
3. Validation of structural variations (SVs) and the graph-based genome……….. ……………..7
4. Comparative genomic analysis across species………………………………..……………8
5. Transcription factor (TF) family analysis……………………………………………...10
6. Transcriptomic, phenotypic, and physiological analyses………………………………………11
    6.1 RNA-seq analysis…………………………………………………………......11
    6.2 Characterization of phenotypic and physiological differences ……………………..……11
7. Contributions of SVs to nearby gene expression and domestication…………………..………12
8. Validation of SVs impacting nearby gene expression………………………………………15
References…………………………………………………………………………16

Supplementary Figures

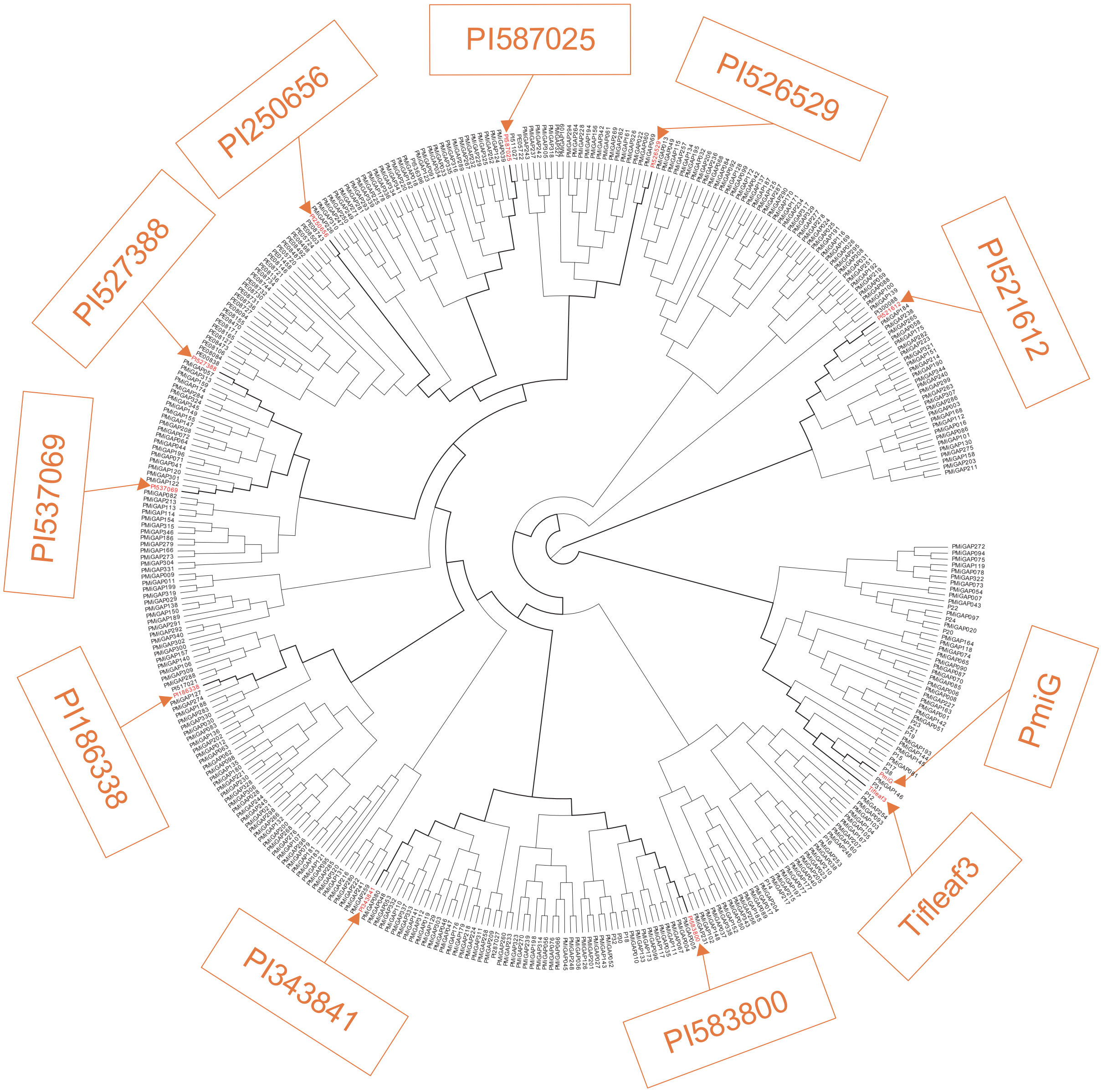

Supplementary Fig. 1 Positions of the 11 pearl millet accessions in the phylogenetic tree of the 394-line collection.

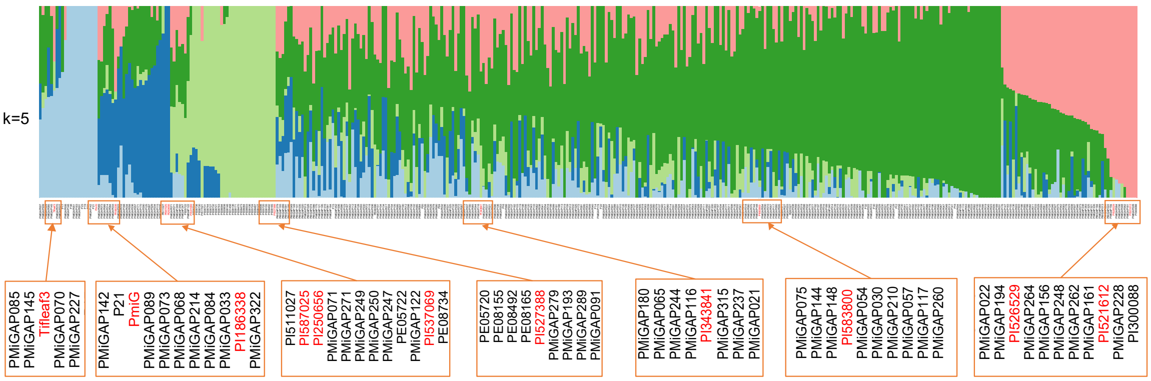

Supplementary Fig. 2 Positions of the 11 pearl millet accessions in a population structural panel. Each accession is represented by a vertical bar, and the length of each colored segment in each bar indicates the proportion contributed by ancestral populations.

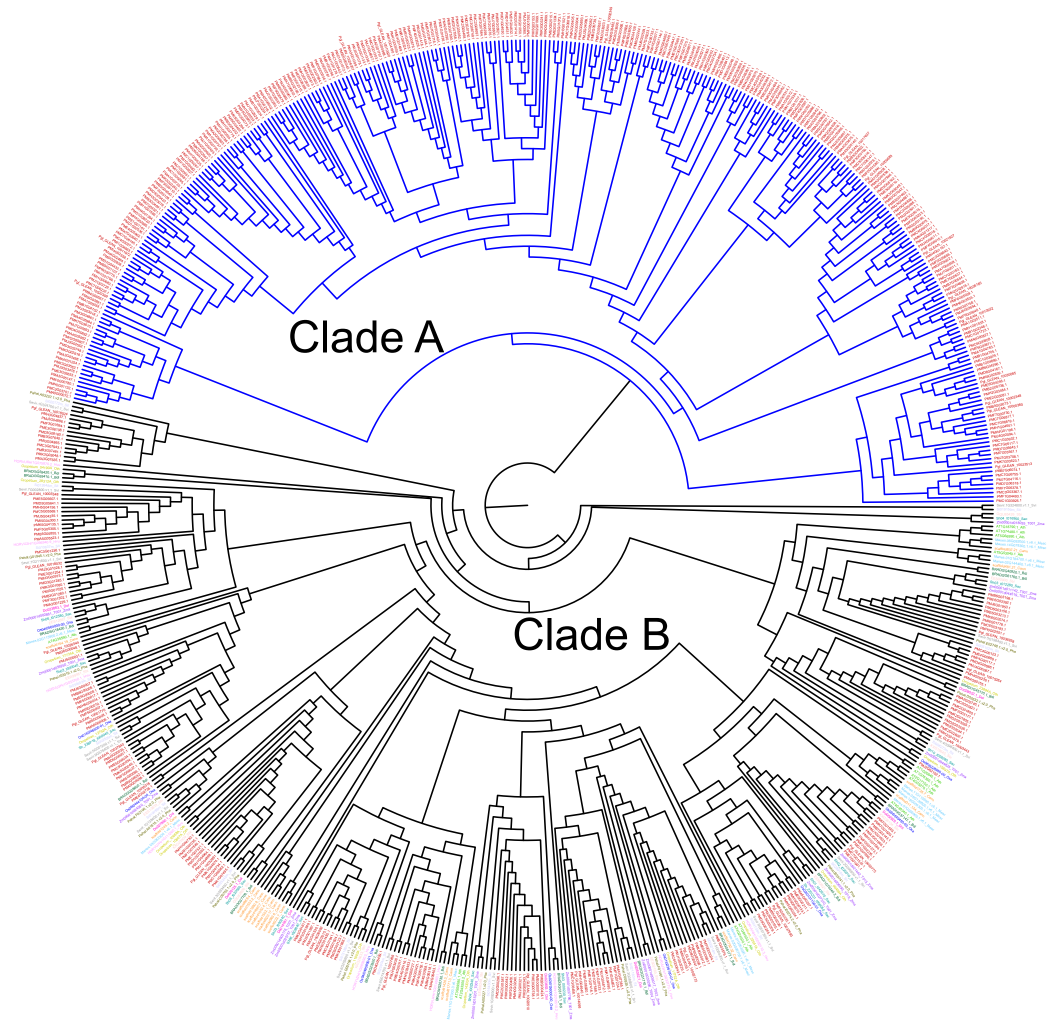

Supplementary Fig. 3 A phylogenetic tree of RWP-RK TFs from pearl millet and the other 14 species shown in Supplementary Table 9.

Supplementary Note

1. Sequencing, assembly, and annotation of ten pearl millet genomes

Leaves of ten pearl millet accessions were collected for library construction and Illumina, Hi-C, PacBio and Bionano sequencing. For Illumina paired-end sequencing, ~1.5 µg of genomic DNA was extracted from each accession to construct a short insert (350 bp) library using a TruSeq Nano DNA HT Sample Preparation Kit (Illumina) following the manufacturer's instructions. For Hi-C sequencing, the leaves were first fixed with formaldehyde and were then lysed. The HindIII enzyme was used to digest the cross-linked DNA. Sticky ends were biotinylated and proximity-ligated to form chimeric junctions. Finally, Hi-C paired-end libraries were constructed by processing the chimeric fragments representing the original cross-linked long-distance physical interactions. The Illumina paired-end and Hi-C libraries were sequenced to produce 150-bp paired-end reads using the Illumina HiSeq X platform. For PacBio HiFi data, the DNA was used to construct 15-kb-insert-size SMRTbell libraries using a SMRTbell Express Template Prep Kit 2.0 following the manufacturer's protocol (PacBio, CA). The constructed libraries were sequenced using the PacBio Sequel II platform, and HiFi reads were obtained using the CCS tool (v6.0.0; https://github.com/PacificBiosciences/ccs) with the settings 'min-passes=3, min-rq=0.99'. For Bionano optical map data, high-molecular weight DNA was extracted using a Bionano Plant Tissue DNA Isolation Kit (Bionano Genomics) and digested with Nt.BssSI (New England Biolabs). Labeled and stained DNA was next loaded onto a Saphyr Chip for sequencing.

We achieved approximately 11.5- to 28.0-fold coverage with PacBio HiFi sequences for each of the ten accessions including PI537069, PI521612, PI526529, PI587025, PI583800, Tifleaf3, PI186338, PI343841, PI527388, and PI250656. The assembly of the initial contigs showed that the longest N50 was 79.18 Mb for PI526529, while the shortest was 3.10 Mb for PI527388 (Table 1). The contigs of PI537069 were further processed using hybrid assembly by integrating high-resolution optical mapping data (BioNano Genomics Irys). Next, chromosome-scale assemblies were constructed *via* Hi-C interaction pairs, which resulted in the anchoring of 96.68% and 95.00% of the assembled bases onto seven chromosomes for PI537069 and Tifleaf3, respectively (Extended Data Fig. 1a,b and Table 1). Subsequently, we performed genome collinearity analysis using the PI537069 chromosome-scale assembly as a reference and observed high chromosome collinearity with Tifleaf3 and with an additional four contig-level assemblies (Fig. 1b and Extended Data Fig. 1c,d), which provided an opportunity to upgrade the contig-level assemblies

to chromosome-level assemblies. Based on these results, the contigs of the other eight accessions were clustered and oriented to obtain chromosome-level assemblies using the PI537069 assembly as a reference. We further randomly assessed contig connections and found several cases in which breakpoints between two contigs could be connected by a long PacBio HiFi read (Extended Data Fig. 1e), indicating that the contigs were accurately ordered. Ultimately, the ten pearl millet genomes had scaffold N50 values ranging from 193.80-286.98 Mb and genome sizes of 1.89-2.00 Gb (Table 1). The HiFi reads were developed recently and obtained with a PacBio Sequel System in circular consensus sequencing (CCS) mode. This strategy can achieve over 99.9% single-molecule read accuracy, which is comparable to the accuracy of short-read and Sanger sequencing and outperforms long-read sequencing[1]. We therefore obtained ten chromosomal level assemblies with decent contig N50 values (average of 19.62 Mb) compared to previous assemblies from grass based on long reads (< 10 Mb)[2-5].

The ten pearl millet genomes were annotated according to a comprehensive strategy that combined homolog prediction, de novo prediction, and other types of evidence-driven prediction. From the combined transcript data, 35,486-38,076 gene models were identified in these ten genomes. Our gene models had an average coding sequence length of approximately 1 kb and an average of four exons per gene (Supplementary Table 2). More than 99% of the genes were annotated to known proteins in other species based on the database (Supplementary Table 2). For repeat annotation, de novo and homolog predictions were utilized to separately mask repeats in the ten genomes. In total, 1.33-1.45 Gb (70.4-72.5%) of transposable elements (TEs) were identified in these genomes (Supplementary Table 2), which exceeded the published genome size (1.22 Gb), suggesting that our assemblies were more complete than the existing genome. Overall, Class I TEs occupied a higher proportion (66.6-68.5%) of the genome than Class II TEs (2.9-4.6%). In Class I, long terminal repeat (LTR)/Gypsy repeat elements showed the largest proportions (44.0-45.8%), followed by LTR/Copia elements (20.3-21.9%). In Class II, more than half of the TEs were in the CMC family (1.6-2.1%) (Supplementary Table 2).

The LTR contents of ten pearl millet accessions (65.5-67.2%, 1.27/1.94G-1.28/1.91G) were greater than those of closely related species such as *Cenchrus purpureus* (55.8%, 1.06/1.90G)[3], *Panicum hallii* (25.0%, 0.12/0.48G)[6], and *Setaria viridis* (17.9%; 0.07/0.39G)[7]. We further estimated the expansion time of the LTR-TEs to be approximately 0.5 million years ago (Mya) in pearl millet, which was more recent than previously reported expansion times of approximately 1

Mya in *P. hallii*, 2 Mya in *S. viridis*, and 2.5 Mya in *C. purpureus* (Extended Data Fig. 1f). These results suggested that LTR expansion occurred recently in pearl millet and may have contributed to the larger genome size of pearl millet than its relatives. We next analyzed genes with young LTR-TE insertion events (< 0.5 million years ago, Mya) and discovered that 323 genes were significantly enriched in pathways related to environmental adaptation, such as ABC transporters and plant-pathogen interaction pathways (Extended Data Fig. 1g). More than half of these genes (53.6%; 173/323) were differentially expressed in Tifleaf3 under heat treatments according to our RNA-seq data (dataset A) (Supplementary Table 3-4), suggesting that recent LTR expansion (< 0.5 Mya) may have contributed to heat stress tolerance in pearl millet.

2. Analysis of core, dispensable, and private genes

We conducted Gene Ontology (GO) and Kyoto Encyclopedia of Genes and Genomes (KEGG) enrichment analyses of the core, dispensable, and private genes of the developed pan-genome (Extended Data Fig. 4b). For GO analysis, the core genes were enriched mainly in general biological process and function terms such as organic substance and primary metabolic processes and catalytic activity. In contrast, the majority of the enriched terms of dispensable genes were related to signal transducer and receptor activities, which are responsible for plant development and responses to biotic and abiotic stresses[8]. The private genes tended to be enriched in single-organism metabolic processes. In the KEGG enrichment analyses, the core genes were enriched in non-homologous end-joining, photosynthesis, proteasome and one carbon pool by folate pathways, and the dispensable genes were enriched oxidative phosphorylation and glycosphingolipid biosynthesis. In contrast, the private genes were enriched in fatty acid metabolism, degradation, and biosynthesis, which are related to the responses of plants to stress[9].

3. Validation of structural variations (SVs) and the graph-based genome

To demonstrate that SVMU reliably predicted the SVs[10], we also used SyRI (v1.6.3)[11], Assmeblytics[12], and smartie-sv[13] tools to detect SVs. Since Assmeblytics only returns PAV, we compared performance of PAV detection between these three tools and SVMU. Approximately 90% of SVs identified by SVMU could be captured by combined outputs from the other three tools (Supplementary Table 6). To further validate the SVs, we performed a PCR genotyping to

successfully validate all selected SVs including three randomly picked from the SV pool and two mentioned in Fig. 5c and Extended Data Fig. 9e.

To validate the quality of the graph-based genome that could be used for SV genotyping in a population, we called SVs from 394 accessions of a pearl millet population[14]. We used the VG tool (v1.25.0)[15] to do this analysis since it has been successfully applied in several major crops such as soybean[16] and rice[17], and it outperforms many other genotyping tools in a benchmark comparison[15,18]. To further evaluate the reliability of the SV genotyping, we used another mapping tool (HISAT2, v2.2.1[19]) to map the short reads from these accessions to the genome (PI537069) that was used as a reference to build the graph-based genome. For validation of the presence of variations, we checked the breakpoints of mapped reads on the border regions of the SVs. If the reads were clipped such that one end of the reads mapped to the reference and the other end of the read mapped to the inserted sequences, the SVs were defined as true positives. For absence variations, if the clipped reads map to two ends of a deletion region, the SVs were defined as true positives. In total, we successfully validated most SVs with an average of 80.6% of the SVs of each accession (Supplementary Table 7), which was similar to the validation results previously reported for the VG tool[18].

In addition, to verify whether the PAVs identified by SVMU were reliable, we mapped short reads from the nine de novo assemblies to the genome (PI537069), which was also used as a reference to build the graph-based genome. We used the same approach mentioned above to evaluate the resulting performance. In total, we successfully validated an average of 88.5% of the SVs for each accession (Supplementary Table 8).

4. Comparative genomic analysis across species

Gene families were constructed using protein sequences from the 11 pearl millet accessions and their relatives, including *Oryza sativa*, *P. hallii*, *Saccharum officinarum*, *Sorghum bicolor*, *S. viridis*, and *Zea mays*. These sequences of these related species were downloaded from Phytozome 12 (https://phytozome.jgi.doe.gov/pz/portal.html)[20] and NCBI (https://www.ncbi.nlm.nih.gov/). Only the longest transcript in the coding region was selected when multiple transcripts were present for a single gene. Proteins with fewer than 30 amino acids were removed. The protein sequences of all species were subjected to BLAST searches using BLASTP (v2.2.26)[21] with the default E-value of 1e-5. The filtered BLAST results were used to cluster the protein sequences into

paralogous and orthologous groups using Orthofinder (v2.3.1)[22] with the default parameters. Subsequently, the protein sequences from single-copy gene families were aligned with MUSCLE (v3.8.31)[23]. A super-alignment matrix was constructed by concatenating the alignments of each gene family. A phylogenetic tree was built using RAxML (v8.0.19; http://sco.h-its.org/exelixis/web/software/raxml/index.html) using the maximum-likelihood method (bootstrap value of 100) with *O. sativa* as an outgroup. The divergence time of each node on the phylogenetic tree was estimated using the MCMCTree program (v4.5; http://abacus.gene.ucl.ac.uk/software/paml.html) with Phylogenetic Analysis by Maximum Likelihood (PAML) with the parameter settings 'burn-in=10000, sample-number=100000, sample-frequency=2'. The initial time was calibrated based on the TimeTree database, as follows[24] (http://www.timetree.org/): *S. bicolor* and *S. officinarum*, 8-12 Mya; *Z. mays* and *S. bicolor*, 12-16 Mya; *Z. mays* and *P. millet*, 18-30 Mya; *Z. mays* and *O. sativa*, 24-52 Mya; *P. hallii* and *P. millet*, 9-23 Mya.

The adaptations of pearl millet to extreme environmental conditions help it to tolerate diverse hostile environments, particularly under hot conditions[25]. Although most of the genes related to tolerance have not been elucidated in pearl millet, this species shows better resistance than its relatives[26,27], which facilitates the identification of stress-related genes *via* comparative genomic analyses. We compared pearl millet to six close relatives (*S. viridis*, *P. hallii*, *C. purpureus*, *Z. mays*, *S. bicolor*, and *S. officinarum*). The 11 pearl millet accessions were most similar to *S. viridis* and *P. hallii* and they shared a common ancestor with the other three species (*Z. mays*, *S. bicolor*, and *S. officinarum*) (Extended Data Fig. 7a). In an analysis aimed at the further identification of stress-related genes across species, a total of 142 expanded, 68 positively selected, and 329 species-specific gene families were identified in pearl millet. These families were also enriched in some pathways and biological processes regulated by stresses (Extended Data Fig. 7b). For example, expanded families were enriched in the response to extracellular stimulus process and the plant-pathogen interaction and flavonoid biosynthesis pathways which are believed to play a key role in the protection of plants against biotic and abiotic stress[28,29]. A recent study revealed important roles of flavonoid biosynthesis in thermotolerance during the rice reproductive stage[30]. The positively selected families were enriched in response to oxidative stress processes, and ABC transporters and phenylpropanoid biosynthesis pathways which are activated under abiotic stress conditions[31,32]. The families unique to pearl millet were associated with the response to chemical

stimulus, pathogenesis, and cellular response to stimulus processes and the flavonoid biosynthesis and plant-pathogen interaction pathways (Extended Data Fig. 7b). Interestingly, the expanded pearl millet genes were enriched in endoplasmic reticulum (ER) related-processes, indicating that the ER might play an important role in the development of pearl millet.

5. Transcription factor (TF) family analysis

We identified the RWP-RK (https://www.ebi.ac.uk/interpro/entry/pfam/PF02042/) TF family, which has undergone expansion, in the genomes of the 11 pearl millet accessions (Fig. 3b). We investigated LTRs located surrounding (5 kb) the *RWP-RKs* and used a binomial to find LTR-TEs were more likely to be enriched around *RWP-RKs* in pearl millet than in rice, sorghum, and maize (Fig. 3c). In brief, we sampled the same number of *RWP-RKs* overlapping with LTR-TEs and repeated this process 100 times to calculate an average overlap ratio. We performed the exact binomial test by setting this overlap ratio as the hypothesized probability of success, the number of *RWP-RKs* overlapping with LTR-TEs as the number of successes, the number of *RWP-RKs* as the number of trials, and the alternative as ‘greater’. We identified these LTRs expanded earlier in pearl millet than in the other species. These results suggest that early LTR expansion might be associated with RWP-RK family expansion in pearl millet (Fig. 3c,d). We further separated the *RWP-RKs* into two clades (A and B) based on a phylogenetic tree in which clade A contained *RWP-RKs* specific to pearl millet (Supplementary Fig. 3 and Supplementary Table 10). Interestingly, the LTRs located near the specific RWP-RKs exhibited more early expansions than those located near nonspecific *RWP-RKs* (Clade B) in most pearl millet accessions (Extended Data Fig. 7c), indicating that the specific increase in *RWP-RKs* might be related to early LTR expansion. We next characterized ten *RWP-RKs* responding to heat stress in coregulated networks. Pearson correlation analysis revealed that 3,376 genes exhibited a strong expression correlation ($p < 0.05$, $|rho| > 0.8$) with the ten *RWP-RKs*, and 1,327 and 698 of them were predicted to interact with these RWP-RKs based on either the STRING tool or binding site predictions, respectively (Supplementary Table 11)[33]. We also characterized one RWP-RK TF (*PMF0G00024.1*) in a coregulated network (Supplementary Table 11) and utilized dual-luciferase assays to verify that this TF could transactivate two stress-related genes: *PMA2G00541.1*, encoding basic leucine zipper protein 9 associated with heat stress[34], and *PMA6G02031.1*, encoding a sodium transporter

associated with salt stress[35] (Fig. 3g). These connections will be helpful for further revealing the contributions of *RWP-RK* members to the response to heat stress in plants.

6. Transcriptomic, phenotypic, and physiological analyses

6.1 RNA-seq analysis

To further explore the transcriptional changes in pearl millet caused by high temperature, we performed transcriptome sequencing of Tifleaf3 plants under continuous heat stress treatment (dataset A) and six different materials under high-temperature treatment (dataset B) and identified 25,684 and 25,186 differentially expressed genes (DEGs), respectively (Supplementary Table 1). We assessed the expression changes of genes within the ER-related pathways. A total of 540 ER-related genes were identified in pearl millet, and 168-219 genes were differentially expressed in the six materials (31.11-40.65%), 36 of which were simultaneously differentially expressed in all six materials. Three genes encoding the two major proteins calnexin (CNX) and calreticulin (CRT), were upregulated in dataset B (1 h treatment). After CNX/CRT is processed, the remaining incorrectly folded peptide chain enters the ER-associated degradation (ERAD) system to be cleared[36]. The effective operation of the ERAD system is very important for maintaining the normal life activities of cells. In the ERAD system, the misfolded protein is recognized again and binds to binding immunoglobulin protein (BiP, https://www.kegg.jp/entry/K09490)[37]. There were 6 DEGs involved in this process, among which one DEG, encoding HSP40 (https://www.kegg.jp/entry/K09505), which was down-regulated in PI537069, and the remaining DEGs, encoding NEF (https://www.kegg.jp/entry/K04573) (2 DEGs), Bip (2 DEGs) and HSP40 (2 DEGs), were all up-regulated. The identified and modified peptides were transported to the cytoplasm by Protein Disulfide-Isomerase A6 (PDIA, https://www.kegg.jp/entry/K09584) (1 upregulated DEG) and degraded by sHSP (18 DEGs), HSP70 (https://www.ebi.ac.uk/interpro/entry/pfam/PF00012/) (7 DEGs, two of which were down-regulated in PI521612 and PI537069, while the rest were up-regulated.) and HSP90 (https://www.ebi.ac.uk/interpro/entry/pfam/PF00183/) (1 DEG; Supplementary Table 12).

6.2 Characterization of phenotypic and physiological differences

Trait variances among different samples are essential for identifying phenotype-related SVs[17]. According to the distinct phenotypes and physiological indicators of the six accessions under heat

treatment (Fig. 5f and Extended Data Fig. 9i), we separated the accessions into HR (4 accessions) and HS (2 accessions) groups. The phylogenetic tree showed that the two HS accessions were not closely related (Extended Data Fig. 7a), suggesting that similarities of their heat susceptibility characteristics were not caused by a close genetic relationship. We identified 150 gene families that specifically existed in the group of four HR accessions. These families were enriched mainly in abiotic stress-related biological processes such as folate, terpenoid backbone, steroid, and N-glycan biosynthesis[38-40] (Extended Data Fig. 9j). Notably, folate biosynthesis can protect plants against oxidative stress and temperature stress[41]. These results reveal clear phenotypic and physiological differences between the HR and HS accessions, adding to the reliable identification of SVs that contribute to distinct heat tolerance differences.

7. Contributions of SVs to nearby gene expression and domestication

The differences in the transcripts per million (TPM) values [FDR-adjusted p value (q value) < 0.05] of nearby genes were calculated for each accession relative to the reference PI537069 (Fig. 5a). Fisher's exact test was conducted to determine if the SVs were enriched in genes with TPM differences, and the obtained p values were corrected to q values with a cutoff of 0.05. The results showed that SVs were enriched in nearby genes with changes in gene expression in all five accessions (q value < 0.05) (Fig. 5a). To further investigate whether the SVs affecting genes exhibited increased sensitivity to heat stress, we relied on RNA-seq dataset A for both leaf and root tissues under heat treatment (Supplementary Table 1). We calculated the proportion of altered genes (|expression fold change| > 1) among 22,181 genes overlapping with SVs (SV-genes; within 5 kb of SVs) and 13,305 genes not overlapping with SVs (nSV-genes) in leaf and root tissues under heat treatments. Fisher's exact test was used to determine whether the SVs were enriched in genes responsive to heat stress. The results from all eight time points showed that SVs were enriched in DEGs ($p < 0.005$), and SV-genes achieved higher proportion of altered genes relative to the nSV-genes (Fig. 5b and Extended Data Fig. 9a). We analyzed the 22,181 genes located near SVs and found that most of these genes (87,76%; 19,467/22,181) were close to SVs that also overlapped with transposons (TE-SV-genes) (Extended Data Fig. 9b). We further analyzed the transcriptional changes in genes located near the 19,467 TE-SV-genes in response to heat stress and found that TE-SVs were also enriched in DEGs ($p < 0.005$) (Extended Data Fig. 9c,d),

suggesting that TEs might also contribute to gene responsiveness to heat stress, similar to previous findings in crops[42,43].

We further designed a pipeline to identify potential SVs associated with heat-related genes (Extended Data Fig. 9k). Briefly, 1) we distinguished four heat-resistant (HR) and two heat-susceptible (HS) accessions based on their phenotypes and physiological indicators under heat treatments (Fig. 5f and Extended Data Fig. 9i). 2) We considered one scenario that SVs were present in three or all four HR accessions and not in any HS accessions. 3) We further filtered 2,354 SVs nearby 2,769 genes (within 5 kb of SVs) and compared expression of these genes between the accessions with and without the SVs based on Wilcoxon test[44]. 4) We comprehensively collected heat-related genes from canonical heat response pathways derived from literatures, GO, and KEGG pathways[45-48]. In addition, based on iTAK (v1.7a)[49] tool, we collect TFs or transcription regulators (TRs), which would be an essential resource for revealing regulatory roles of these TFs or TRs underlying heat tolerance. 5) We filtered SVs nearby the genes collected in the step 4 and obtained 43 candidate SVs potentially associated with 34 heat-related genes and focused on four focal SVs (Fig. 5g and Supplementary Table 13).

To characterize the SVs underlying heat tolerance during adaptation and domestication in pearl millet, we analyzed a previously released dataset (SRP063925)[14] consisting of 29 improved cultivated, 255 landrace, and 29 wild accessions (Supplementary Table 1). These accessions were representative of the geographical diversity of pearl millet. A total of 17 origins were recorded in this population; almost all of the regions included landrace accessions, and approximately half of them included improved cultivars (Supplementary Table 1). The improved cultivars and landrace accessions both came from Pearl Millet Inbred Germplasm Association Panel (PMiGAP) lines that were developed at ICRISAT in partnership with Aberystwyth University[50]. PMiGAP is a commonly used pearl millet panel that serves as a repository of approximately 29 million genome-wide SNPs and has been used to map many traits, including drought tolerance, grain Fe and Zn contents, nitrogen use efficiency, components of endosperm starch, and grain yield[50]. In this population, we identified a total of 124,532 SVs (minor allele frequency ≥ 0.05; missing rate ≤ 0.1), which were genotyped by mapping all of the re-sequences contributing to the graph-based pan-genome. We subsequently explored potential heat stress adaptation hotspots of SVs in pearl millet through the comparison of the different population classifications. For temperature adaptation, we separated 191 accessions with known latitude information into two groups,

originating from tropical (23°27' N-23°27' S) and temperate zones (66°33'-23°27' N; 23°27'-66°33' S)[14,51] (Supplementary Table 1). We focused on the SVs with population frequency differences (fdSVs) between these two groups by applying sliding window methodology[52]. We further correlated the latitudes of their origins with the presence or absence of 20 heat tolerance-related fdSVs across the 191 genotypes and identified one fdSV that was significantly associated with accessions from higher latitudes and another fdSV associated with accessions from lower latitudes (Fig. 6a and Extended Data Fig. 10b).

In pearl millet, some domestication-related loci and candidate genes for significant traits, such as grain number per panicle (GNP), have been found to be common across years according to SNP-based genome-wide association studies (GWASs)[14]. However, this method is limited to clarify the potential mechanisms underlying candidate gene regulation. To explore the utility of the graph-based genome and identify SV-driven alterations of genes controlling important agronomic traits, we performed GWAS using 124,532 PAVs and 1,455,924 SNPs in 242 accessions from PMiGAP lines[14] based on a mixed linear model (MLM)[53]. In total, we identified 201 significant associations including 142 PAVs associated with 20 traits (Supplementary Table 19). To technically validate the association results, we used the 'LightGBM' tool based on a gradient boosting learning paradigm that addresses the population stratification issue well[54]. We identified most associations (87.6%; 176/201) that could be revealed based on the 'LightGBM' method (Supplementary Table 19). We next focused on the selection trait GNP, and found an association peak on chromosome 5 that overlapped between PAVs and SNPs[14]. This peak region contained 8 significant SVs located near 12 candidate genes (Fig. 6c). To further examine whether this association is stable under different conditions, we additionally conducted PAV-GWAS under stressful and field conditions over two years (2011 and 2012). A strong association at this QTL was observed under both conditions in both years (Extended Data Fig. 10e). In this peak, we also identified 14 PAVs associated with grain number/m^2 (GNM2) under at least one condition. Among these SVs, one LTR/Gypsy deletion was located nearby genes and positioned 30.7 kb upstream of *PMA5G04389.1*, which is orthologous to *CYP71B16* (https://www.ncbi.nlm.nih.gov/gene/822215) of *Arabidopsis* (Extended Data Fig. 10f). This gene was reported to be co-expressed with *CYP78A9* (https://www.ncbi.nlm.nih.gov/gene/825361), which is involved in reproductive development[55]. We observed this deletion in 29 accessions showing lower GNM2 values relative to 213 accessions without the deletion (Extended Data Fig. 10g and Supplementary Table 19). In

addition, we analyzed the Till trait (tiller number/plant), which is also critical for grain yield, and found four significantly associated SVs near six genes that were not identified based on the SNP data (Extended Data Fig. 10h), revealing additional hidden genetic variations that may not be represented by SNPs.

8. Validation of SVs impacting nearby gene expression

We further focused on two important genes involved in the HSR (Supplementary Table 13). One of these genes (*PMA5G04793.1*) was orthologous to *AT1G52730*, involved in plant target of rapamycin (TOR) signaling, which is essential for cells to sense stressful conditions[56]. This gene harbored a 260-bp DEL in the upstream regulatory region only in the HR group with low expression (Fig. 5c). The other gene (*PMA6G05740.1*) was orthologous to *AT5G43130*, which encodes a transcription initiation factor TFIID subunit 4B protein (TAF4, https://www.ncbi.nlm.nih.gov/gene/834330) and had a 1,321-bp DEL in the promoter region in all HR accessions (Extended Data Fig. 9e). The *TAF4* gene shares a conserved RCD1-SRO-TAF4 (RST) domain with the radical-induced cell death1 (RCD1, https://www.ncbi.nlm.nih.gov/gene/840115) protein, which is an important regulator of stress responses in plants[57]. These two DELs could potentially cause lower gene expression in the HR group than in the HS group, and this assumption was confirmed by a transient gene expression experiment in tobacco (*Nicotiana tabacum*) leaves (Fig. 5d,e and Extended Data Fig. 9f,g). More specifically, when the *PMA5G04793.1* promoter was transformed into tobacco leaves, the GUS phenotype results showed that the part of the leaf transformed with SVs did not show histochemical staining, unlike the part not transformed with SVs (-SV) (Fig. 5d). The normal promoter sequence of the gene (promoter [-SV]) showed higher GUS activity than the promoter of the gene with the SV (promoter [+SV]) in tobacco leaves (Fig. 5e), suggesting that the normal promoter (-SV) resulted in more GUS protein activation. This result indicates that SVs can influence downstream gene expression. Regarding the transformation of the *PMA6G05740.1* promoter, we found that the part of the leaf without SV transformation displayed no staining under heat stress, unlike the part transformed with SVs (Extended Data Fig. 9f). The normal promoter sequence of the gene (promoter [-SV]) showed lower GUS activity than the promoter of the gene with the SV (promoter [+SV]) (Extended Data Fig. 9g), which was consistent with the observation that *PMA6G05740.1*, near this SV, exhibited more downregulation in the HS (-SV) group than in the HR (+SV) group

under heat treatment (Extended Data Fig. 9e). This result suggests that SVs may inhibit the downregulation of nearby genes under heat stress.

References

1. Hon, T. *et al.* Highly accurate long-read HiFi sequencing data for five complex genomes. *Sci. Data* **7**, 1-11 (2020).
2. Huang, L. *et al.* Genome assembly provides insights into the genome evolution and flowering regulation of orchardgrass. *Plant Biotechnol. J.* **18**, 373-388 (2020).
3. Yan, Q. *et al.* The elephant grass (*Cenchrus purpureus*) genome provides insights into anthocyanidin accumulation and fast growth. *Mol. Ecol. Resour.* **21**, 526-542 (2021).
4. Zhang, G. *et al.* The reference genome of *Miscanthus floridulus* illuminates the evolution of Saccharinae. *Nat. Plants* **7**, 608-618 (2021).
5. Lovell, J.T. *et al.* Genomic mechanisms of climate adaptation in polyploid bioenergy switchgrass. *Nature* **590**, 438-444 (2021).
6. Lovell, J.T. *et al.* The genomic landscape of molecular responses to natural drought stress in Panicum hallii. *Nat Commun.* **9**, 1-10 (2018).
7. Mamidi, S. *et al.* A genome resource for green millet *Setaria viridis* enables discovery of agronomically valuable loci. *Nat. Biotechnol.* **38**, 1203-1210 (2020).
8. Peck, S. & Mittler, R. Plant signaling in biotic and abiotic stress. (Oxford University Press UK, 2020).
9. Upchurch, R.G. Fatty acid unsaturation, mobilization, and regulation in the response of plants to stress. *Biotechnol. Lett.* **30**, 967-977 (2008).
10. Marçais, G. *et al.* MUMmer4: a fast and versatile genome alignment system. *PLoS Comp. Biol.* **14**, 1-14 (2018).
11. Goel, M., Sun, H., Jiao, W.-B. & Schneeberger, K. SyRI: finding genomic rearrangements and local sequence differences from whole-genome assemblies. *Genome Biol.* **20**, 1-13 (2019).
12. Nattestad, M. & Schatz, M.C. Assemblytics: a web analytics tool for the detection of variants from an assembly. *Bioinformatics* **32**, 3021-3023 (2016).
13. Kronenberg, Z.N. *et al.* High-resolution comparative analysis of great ape genomes. *Science* **360**, eaar6343 (2018).
14. Varshney, R.K. *et al.* Pearl millet genome sequence provides a resource to improve agronomic traits in arid environments. *Nat. Biotechnol.* **35**, 969-976 (2017).
15. Garrison, E. *et al.* Variation graph toolkit improves read mapping by representing genetic variation in the reference. *Nat. Biotechnol.* **36**, 875-879 (2018).
16. Liu, Y. *et al.* Pan-genome of wild and cultivated soybeans. *Cell* **182**, 162-176. e13 (2020).
17. Qin, P. *et al.* Pan-genome analysis of 33 genetically diverse rice accessions reveals hidden genomic variations. *Cell* **184**, 3542-3558 (2021).
18. Hickey, G. *et al.* Genotyping structural variants in pangenome graphs using the vg toolkit. *Genome Biol.* **21**, 1-17 (2020).
19. Kim, D., Langmead, B. & Salzberg, S.L. HISAT: a fast spliced aligner with low memory requirements. *Nat. Methods* **12**, 357-360 (2015).

20. Goodstein, D.M. *et al.* Phytozome: a comparative platform for green plant genomics. *Nucleic Acids Res.* **40**, D1178-D1186 (2012).
21. Altschul, S.F., Gish, W., Miller, W., Myers, E.W. & Lipman, D.J. Basic local alignment search tool. *J. Mol. Biol.* **215**, 403-410 (1990).
22. Emms, D.M. & Kelly, S. OrthoFinder: phylogenetic orthology inference for comparative genomics. *Genome Biol.* **20**, 1-14 (2019).
23. Edgar, R.C. MUSCLE: multiple sequence alignment with high accuracy and high throughput. *Nucleic Acids Res.* **32**, 1792-1797 (2004).
24. Kumar, S., Stecher, G., Suleski, M. & Hedges, S.B. TimeTree: a resource for timelines, timetrees, and divergence times. *Mol. Biol. Evol.* **34**, 1812-1819 (2017).
25. Pucher, A. *et al.* Agro-morphological characterization of West and Central African pearl millet accessions. *Crop Sci.* **55**, 737-748 (2015).
26. Ashraf, M. & Hafeez, M. Thermotolerance of pearl millet and maize at early growth stages: growth and nutrient relations. *Biol. Plant.* **48**, 81-86 (2004).
27. Zegada-Lizarazu, W. & Iijima, M. Deep root water uptake ability and water use efficiency of pearl millet in comparison to other millet species. *Plant Prod. Sci.* **8**, 454-460 (2005).
28. Commisso, M. *et al.* Impact of phenylpropanoid compounds on heat stress tolerance in carrot cell cultures. *Front. Plant Sci.* **7**, 1439 (2016).
29. Mierziak, J., Kostyn, K. & Kulma, A. Flavonoids as important molecules of plant interactions with the environment. *Molecules* **19**, 16240-16265 (2014).
30. Cai, Z. *et al.* Transcriptomic analysis reveals important roles of lignin and flavonoid biosynthetic pathways in rice thermotolerance during reproductive stage. *Front. Genet.* **11**, 1120 (2020).
31. Sharma, A. *et al.* Response of phenylpropanoid pathway and the role of polyphenols in plants under abiotic stress. *Molecules* **24**, 2452 (2019).
32. Moon, S. & Jung, K.-H. Genome-wide expression analysis of rice ABC transporter family across spatio-temporal samples and in response to abiotic stresses. *J. Plant Physiol.* **171**, 1276-1288 (2014).
33. Snel, B., Lehmann, G., Bork, P. & Huynen, M.A. STRING: a web-server to retrieve and display the repeatedly occurring neighbourhood of a gene. *Nucleic Acids Res.* **28**, 3442-3444 (2000).
34. Wang, L. *et al.* Differential physiological, transcriptomic and metabolomic responses of Arabidopsis leaves under prolonged warming and heat shock. *BMC Plant Biol.* **20**, 1-15 (2020).
35. Chu, M., Chen, P., Meng, S., Xu, P. & Lan, W. The *Arabidopsis* phosphatase PP2C49 negatively regulates salt tolerance through inhibition of AtHKT1; 1. *J. Integr. Plant Biol.* **63**, 528-542 (2021).
36. Parodi, A.J. Protein glucosylation and its role in protein folding. *Annu. Rev. Biochem.* **69**, 69-93 (2000).
37. Ushioda, R., Hoseki, J. & Nagata, K. Glycosylation-independent ERAD pathway serves as a backup system under ER stress. *Mol. Biol. Cell* **24**, 3155-3163 (2013).
38. Strasser, R. Biological significance of complex N-glycans in plants and their impact on plant physiology. *Front. Plant Sci.* **5**, 363 (2014).

39. Salchert, K., Bhalerao, R., Koncz–Kálmán, Z. & Koncz, C. Control of cell elongation and stress responses by steroid hormones and carbon catabolic repression in plants. *Philos. Trans. R. Soc. Lond., Ser. B: Biol. Sci.* **353**, 1517-1520 (1998).
40. Basyuni, M. *et al.* Expression of terpenoid synthase mRNA and terpenoid content in salt stressed mangrove. *J. Plant Physiol.* **166**, 1786-1800 (2009).
41. Xiang, N. *et al.* Effects of temperature stress on the accumulation of ascorbic acid and folates in sweet corn (*Zea mays* L.) seedlings. *J. Sci. Food Agric.* **100**, 1694-1701 (2020).
42. Makarevitch, I. *et al.* Transposable elements contribute to activation of maize genes in response to abiotic stress. *PLoS Genet.* **11**, e1004915 (2015).
43. Naito, K. *et al.* Dramatic amplification of a rice transposable element during recent domestication. *PNAS* **103**, 17620-17625 (2006).
44. Bauer, D.F. Constructing confidence sets using rank statistics. *J. Am. Stat. Assoc.* **67**, 687-690 (1972).
45. Ohama, N., Sato, H., Shinozaki, K. & Yamaguchi-Shinozaki, K. Transcriptional regulatory network of plant heat stress response. *Trends Plant Sci.* **22**, 53-65 (2017).
46. Zhang, H., Zhu, J., Gong, Z. & Zhu, J.-K. Abiotic stress responses in plants. *Nat. Rev. Genet.* **23**, 104-119 (2022).
47. Gil, K.E. & Park, C.M. Thermal adaptation and plasticity of the plant circadian clock. *New Phytol.* **221**, 1215-1229 (2019).
48. Ding, Y., Shi, Y. & Yang, S. Molecular regulation of plant responses to environmental temperatures. *Mol. Plant* **13**, 544-564 (2020).
49. Zheng, Y. *et al.* iTAK: a program for genome-wide prediction and classification of plant transcription factors, transcriptional regulators, and protein kinases. *Mol. Plant* **9**, 1667-1670 (2016).
50. Srivastava, R.K. *et al.* Genome-wide association studies and genomic selection in Pearl Millet: Advances and prospects. *Front. Genet.*, 1389 (2020).
51. Wilde, S.P.R. & Mulholland, P. Return to Earth: a new mathematical model of the Earth's climate. *Int. J. Atmos. Oce. Sci.* **4**, 36-53 (2020).
52. Chen, H., Patterson, N. & Reich, D. Population differentiation as a test for selective sweeps. *Genome Res.* **20**, 393-402 (2010).
53. Zhou, X. & Stephens, M. Genome-wide efficient mixed-model analysis for association studies. *Nat. Genet.* **44**, 821-824 (2012).
54. Yan, J. *et al.* LightGBM: Accelerated genomically designed crop breeding through ensemble learning. *Genome Biol.* **22**, 1-24 (2021).
55. Sotelo-Silveira, M. *et al.* Cytochrome P450 CYP78A9 is involved in Arabidopsis reproductive development. *Plant Physiol.* **162**, 779-799 (2013).
56. Van Leene, J. *et al.* Capturing the phosphorylation and protein interaction landscape of the plant TOR kinase. *Nat. Plants* **5**, 316-327 (2019).
57. Jaspers, P., Brosché, M., Overmyer, K. & Kangasjär, J. The transcription factor interacting protein RCD1 contains a novel conserved domain. *Plant Signal. Behav.* **5**, 78-80 (2010).
