# Supplementary material for: Pangenomic analysis identifies structural variation associated with heat tolerance in pearl millet
Source: Nat Genet. 2023 Mar 2;55(3):507–18. doi: 10.1038/s41588-023-01302-4 (PMC10011142; doi:10.1038/s41588-023-01302-4)
Supplement: Supplementary file 2 — Reporting Summary [file 41588_2023_1302_MOESM2_ESM.pdf]

nature portfolio

Corresponding author(s): Linkai Huang, Shilin Tian

Last updated by author(s): Jan 11, 2023

Reporting Summary

Nature Portfolio wishes to improve the reproducibility of the work that we publish. This form provides structure for consistency and transparency in reporting. For further information on Nature Portfolio policies, see our Editorial Policies and the Editorial Policy Checklist.

Statistics

For all statistical analyses, confirm that the following items are present in the figure legend, table legend, main text, or Methods section.

n/a	Confirmed	
☐	☒	The exact sample size (*n*) for each experimental group/condition, given as a discrete number and unit of measurement
☒	☐	A statement on whether measurements were taken from distinct samples or whether the same sample was measured repeatedly
☐	☒	The statistical test(s) used AND whether they are one- or two-sided<br>*Only common tests should be described solely by name; describe more complex techniques in the Methods section.*
☒	☐	A description of all covariates tested
☒	☐	A description of any assumptions or corrections, such as tests of normality and adjustment for multiple comparisons
☐	☒	A full description of the statistical parameters including central tendency (e.g. means) or other basic estimates (e.g. regression coefficient) AND variation (e.g. standard deviation) or associated estimates of uncertainty (e.g. confidence intervals)
☐	☒	For null hypothesis testing, the test statistic (e.g. *F*, *t*, *r*) with confidence intervals, effect sizes, degrees of freedom and *P* value noted<br>*Give P values as exact values whenever suitable.*
☐	☒	For Bayesian analysis, information on the choice of priors and Markov chain Monte Carlo settings
☒	☐	For hierarchical and complex designs, identification of the appropriate level for tests and full reporting of outcomes
☐	☒	Estimates of effect sizes (e.g. Cohen's *d*, Pearson's *r*), indicating how they were calculated

Our web collection on statistics for biologists contains articles on many of the points above.

Software and code

Policy information about availability of computer code

Data collection	No software was used in data collection.
Data analysis	Flow cytometry analysis: Kaluza (v2.1.3).<br>Initial assembly: Hifiasm package (v0.13-r308), Pruge_haplotig (v1.1.0), Bionano Solve (v3.5.1).<br>Pseudochromosome construction: BWA (v0.7.8), ALLHIC package (v0.9.8).<br>Genome assessment: BUSCO (v4.1.2), CEGMA (v2.5), BWA (v0.7.8), Merqury (v1.3), LTR_retriever (v2.8).<br>Annotation of repetitive sequences: RepeatMasker (v4.0.5), LTR_FINDER (v1.0.7), Piler (v3.3.0), RepeatScout (v1.0.5), RepeatModeler (v1.0.8), MUSCLE (v3.8.31).<br>Annotation of gene structure: TblastN (v2.2.26), Solar (v0.9.6), GeneWise (v2.4.1), TopHat (v2.0.13), Cufflinks (v2.1.1), Trinity (v2.1.1), PASA, Augustus (v3.2.3), GENSCAN (v1.0), GlimmerHMM (v3.0.1), EVidenceModeler (v1.1.1), SNAP (v2013.11.29), geneid (v1.4).<br>Functional annotation of protein-coding genes: InterProScan (v4.8), HMMER (v3.1), InterPro (v32.0), Pfam (v27.0).<br>Comparative genomic analysis across species: BLASTP (v2.2.26), Orthofinder (v2.3.1), MUSCLE (v3.8.31), RAxML (v8.0.19), MCMCTree program (v4.5).<br>Pan-genome construction: Orthofinder (v2.3.1).<br>SV identification: MUMmer (v4.0.0), SyRI (v1.6.3), vg (v1.25.0).<br>Validation of structural variations: SyRI (v1.6.3), Assmeblytics, smartie-sv, vg (v1.25.0), HISAT2 (v2.2.1).<br>TF family identification and analysis: Fimo (v5.3.2), iTAK tool (v1.7 a).<br>PAV-GWAS: GEMMA (v0.94.1), LightGBM.<br>RNA-seq: FastQC (v0.11.9), Kallisto (v0.46.2), DESeq2 (1.26.0).

For manuscripts utilizing custom algorithms or software that are central to the research but not yet described in published literature, software must be made available to editors and reviewers. We strongly encourage code deposition in a community repository (e.g. GitHub). See the Nature Portfolio guidelines for submitting code & software for further information.

Data

Policy information about availability of data

All manuscripts must include a data availability statement. This statement should provide the following information, where applicable:

- Accession codes, unique identifiers, or web links for publicly available datasets
- A description of any restrictions on data availability
- For clinical datasets or third party data, please ensure that the statement adheres to our policy

The raw sequencing data and transcriptome data of PI186338, PI250656, PI343841, PI521612, PI526529, PI527388, PI537069, PI583800, PI587025, and Tifleaf3 have been deposited in the NCBI Sequence Read Archive under BioProject accession numbers PRJNA749489, PRJNA689619, and PRJNA756390. The assemblies of ten pearl millet have been deposited in NCBI GenBank under the accession numbers JAMZRY000000000 (PI343841), JAMOAQ000000000 (PI250656), JAMKQL000000000 (PI186338), JAMKQK000000000 (PI527388), JAJHQD000000000 (PI587025), JAIFIR000000000 (PI537069), JAINUP000000000 (Tifleaf3), JAINUO000000000 (PI583800), JAINUN000000000 (PI526529), and JAINUM000000000 (PI521612). These assemblies are also available at a website (http://117.78.45.2:91/download). The raw genome assembly data are available under accession number PRJNA749489. The transcriptomic data are available under accession numbers PRJNA749489, PRJNA689619, and PRJNA756390. The public RNA-seq data used was downloaded from NCBI and the bioproject accession numbers is PRJNA520822. The public re-sequence data used was downloaded from NCBI and the accession number is SRP063925. Source data are provided with this paper.

Human research participants

Policy information about studies involving human research participants and Sex and Gender in Research.

Reporting on sex and gender	not applicable
Population characteristics	not applicable
Recruitment	not applicable
Ethics oversight	not applicable

Note that full information on the approval of the study protocol must also be provided in the manuscript.

Field-specific reporting

Please select the one below that is the best fit for your research. If you are not sure, read the appropriate sections before making your selection.

☒ Life sciences ☐ Behavioural & social sciences ☐ Ecological, evolutionary & environmental sciences

For a reference copy of the document with all sections, see nature.com/documents/nr-reporting-summary-flat.pdf

Life sciences study design

All studies must disclose on these points even when the disclosure is negative.

Sample size	Bionano: 1 sample; Hi-C: 2 samples; Pacbio HiFi: 10 samples; Illumina: 228 samples. no sample size calculation was performed. We built the pan-genome based on 11 representative accessions where 10 samples are de-novo assembled in our study and one sample downloaded from a published study. We used Bionano and Hi-C sequencing for PI537069 accession, aiming to obtain a high-quality assemble that could be used as the reference genome for the SV discoveries in the downstream analysis. For the 228 samples of Illumina sequencing, we did bulk RNA-seq analyses including leaf and root tissues and eight time points underlying heat stressful conditions (Supplementary Table 1: Overview of RNA-seq).
Data exclusions	For PAV-GWAS we excluded samples without phenotype data. For temperature adaptation analyses, we excluded samples without latitude data.
Replication	Three biological and three technical replicates for Dual-luciferase assays. Two biological and one technical replicates for Tobacco leaf transformation assays. One biological and technical replicate for PCR validation. Three biological replicates for physiological analysis. Two replicates for flow cytometry.
Randomization	Plants were randomly allocated in the greenhouse. tobacco leaves were randomly collected from individuals with same growth stages. For evaluation of contig connections, we randomly picked several to present in the Extended Fig. 1e. To further validate the SVs, we performed a PCR genotyping to validate three SVs randomly picked from the SV pool. For RNA-seq, the leaves or roots of 16 seedlings with consistent growth were randomly selected and stored in cryogenic vials. For Physiological indicators, the leaves of plants with consistent growth were randomly selected and stored in cryogenic vials.
Blinding	The experiments were conducted blindly. All genotypes were only labeled by numbers when planting, so the investigators did not know the

Blinding | exact accession names

Reporting for specific materials, systems and methods

We require information from authors about some types of materials, experimental systems and methods used in many studies. Here, indicate whether each material, system or method listed is relevant to your study. If you are not sure if a list item applies to your research, read the appropriate section before selecting a response.

Materials & experimental systems

n/a	Involved in the study
☒	☐ Antibodies
☒	☐ Eukaryotic cell lines
☒	☐ Palaeontology and archaeology
☒	☐ Animals and other organisms
☒	☐ Clinical data
☒	☐ Dual use research of concern

Methods

n/a	Involved in the study
☒	☐ ChIP-seq
☐	☒ Flow cytometry
☒	☐ MRI-based neuroimaging

Flow Cytometry

Plots

Confirm that:

☒ The axis labels state the marker and fluorochrome used (e.g. CD4-FITC).

☒ The axis scales are clearly visible. Include numbers along axes only for bottom left plot of group (a 'group' is an analysis of identical markers).

☐ All plots are contour plots with outliers or pseudocolor plots.

☐ A numerical value for number of cells or percentage (with statistics) is provided.

Methodology

Sample preparation	About 20mg leaves, add 1ml MGB, add 500µl lysis buffer, 25µl 50µg/ml PI and 25µl 50µg/ml RNase, mix and shade before use.
Instrument	Beckman CytoFLEX.
Software	CytExpert (version:2.3.0.84).
Cell population abundance	CytoFLEX flow cytometer automatically collects cells and counts the number.
Gating strategy	Use FSC-A/SSC-A to select cells, use PE-A/PE-H to exclude cell debris, and select the location of the positive result of PI staining.

☒ Tick this box to confirm that a figure exemplifying the gating strategy is provided in the Supplementary Information.
