# Supplementary material for: Pangenomic analysis identifies structural variation associated with heat tolerance in pearl millet
Source: Nat Genet. 2023 Mar 2;55(3):507–18. doi: 10.1038/s41588-023-01302-4 (PMC10011142; doi:10.1038/s41588-023-01302-4)
Supplement: Source Data Extended Data Fig. 3 — Source data from the flow cytometry analysis. [file 41588_2023_1302_MOESM4_ESM.pdf]

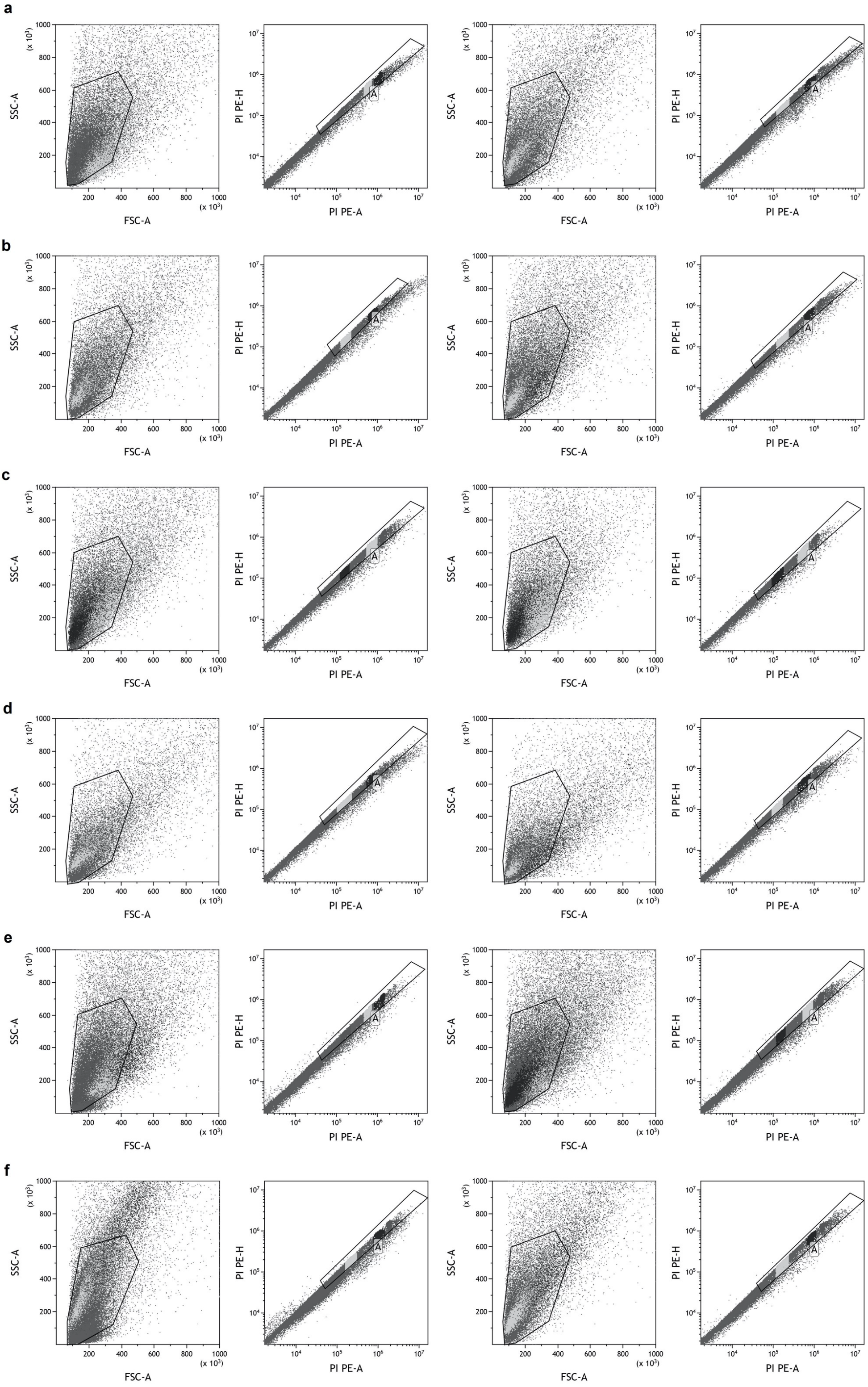

Source Data Extended Data Fig. 3 (a-f) The gating strategy of PI186338 (a), PI250656 (b), PI343841 (c), PI527388 (d) with two biological repeats, corresponding to Extended Data Fig.3. SSC: Lateral scattered light signal; FSC: Forward scattered light signal; PE-A: Relative fluorescence intensities of PI-stained nuclei.

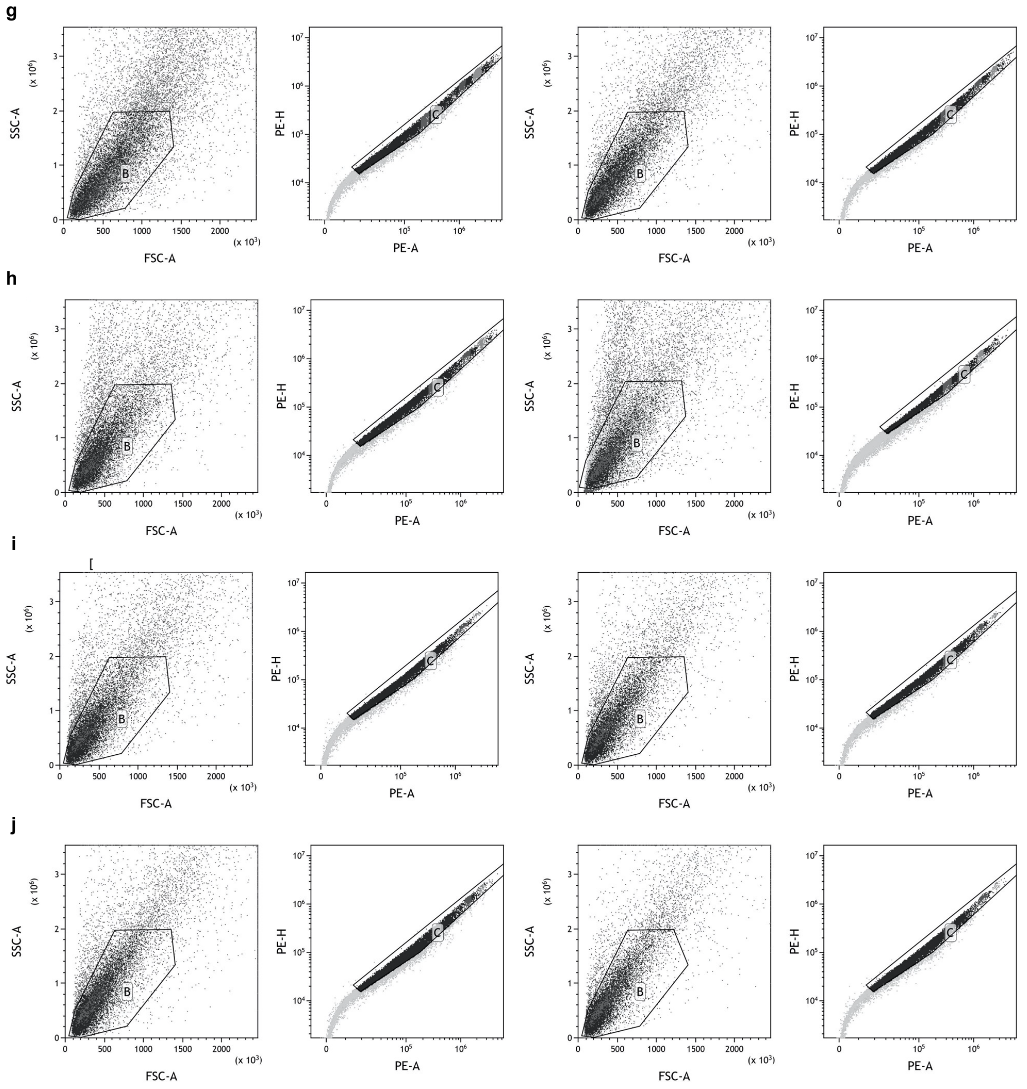

Source Data Extended Data Fig. 3 (g-j) The gating strategy of PI186338 (g), PI250656 (h), PI343841 (i),PI527388 (j) with two biological repeats, corresponding to Extended Data Fig. 3. SSC: Lateral scattered light signal; FSC: Forward scattered light signal; PE-A: Relative fluorescence intensities of PI-stained nuclei.
