# Supplementary material for: Pangenomic analysis identifies structural variation associated with heat tolerance in pearl millet
Source: Nat Genet. 2023 Mar 2;55(3):507–18. doi: 10.1038/s41588-023-01302-4 (PMC10011142; doi:10.1038/s41588-023-01302-4)
Supplement: Source Data Extended Data Fig. 9 — Unprocessed gels. [file 41588_2023_1302_MOESM6_ESM.pdf]

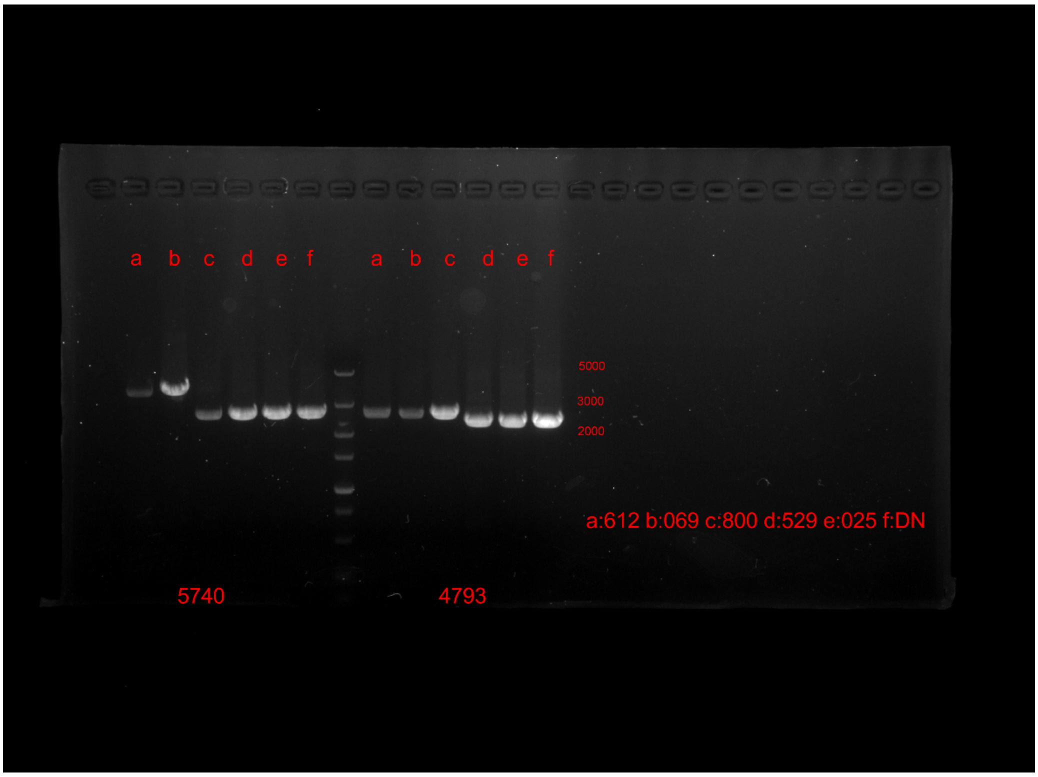

Source Data Extended Data Fig. 9 The uncropped scan of a gel plot corresponding to Extended Data Fig. 9h.
